# Abundant Intergenic TAACTGA Direct Repeats and Putative Alternate RNA Polymerase β′ Subunits in Marine *Beggiatoaceae* Genomes: Possible Regulatory Roles and Origins

**DOI:** 10.3389/fmicb.2015.01397

**Published:** 2015-12-16

**Authors:** Barbara J. MacGregor

**Affiliations:** Department of Marine Sciences, University of North Carolina–Chapel HillChapel Hill, NC, USA

**Keywords:** heptamer repeats, DNA-directed RNA polymerase, beta prime subunit, *Beggiatoaceae*, Cyanobacteria, Bacteroidetes, orange Guaymas “Maribeggiatoa”

## Abstract

The genome sequences of several giant marine sulfur-oxidizing bacteria present evidence of a possible post-transcriptional regulatory network that may have been transmitted to or from two distantly related bacteria lineages. The draft genome of a *Cand*. “Maribeggiatoa” filament from the Guaymas Basin (Gulf of California, Mexico) seafloor contains 169 sets of TAACTGA direct repeats and one indirect repeat, with two to six copies per set. Related heptamers are rarely or never found as direct repeats. TAACTGA direct repeats are also found in some other *Beggiatoaceae, Thiocystis violascens*, a range of Cyanobacteria, and five Bacteroidetes. This phylogenetic distribution suggests they may have been transmitted horizontally, but no mechanism is evident. There is no correlation between total TAACTGA occurrences and repeats per genome. In most species the repeat units are relatively short, but longer arrays of up to 43 copies are found in several Bacteroidetes and Cyanobacteria. The majority of TAACTGA repeats in the *Cand*. “Maribeggiatoa” Orange Guaymas (BOGUAY) genome are within several nucleotides upstream of a putative start codon, suggesting they may be binding sites for a post-transcriptional regulator. Candidates include members of the ribosomal protein S1, Csp (cold shock protein), and Csr (carbon storage regulator) families. No pattern was evident in the predicted functions of the open reading frames (ORFs) downstream of repeats, but some encode presumably essential products such as ribosomal proteins. Among these is an ORF encoding a possible alternate or modified RNA polymerase beta prime subunit, predicted to have the expected subunit interaction domains but lacking most catalytic residues. A similar ORF was found in the *Thioploca ingrica* draft genome, but in no others. In both species they are immediately upstream of putative sensor kinase genes with nearly identical domain structures. In the marine *Beggiatoaceae*, a role for the TAACTGA repeats in translational regulation is suggested. More speculatively, the putative alternate RNA polymerase subunit could be a negative transcriptional regulator.

## Introduction

Organic-rich sediments surrounding hydrothermal sites on the Guaymas Basin sea floor often host luxuriant microbial mats, visually dominated by large filamentous, vacuolated, orange-pigmented, and unpigmented *Beggiatoaceae* (Jannasch et al., 1989). From 16S rRNA data, these appear to belong to several distinct species. None of them are yet in culture, but physiological (McHatton et al., 1996) and genomic (MacGregor et al., 2013a) studies are consistent with a sulfur-oxidizing, nitrate-reducing metabolism. They are gradient dwellers, living between hot sulfidic fluids flowing up through the sediments below and cold, oxygenated overlying seawater. In general, the pigmented forms are found toward the center of mats, where flow rates (and temperature) are higher, while unpigmented forms are more concentrated at the periphery (McKay et al., 2012). The pigmentation is thought to be due to high concentrations of an octaheme cytochrome, possibly a nitrite reductase (MacGregor et al., 2013b). The Orange Guaymas *Cand*. “Maribeggiatoa” (BOGUAY) draft genome (MacGregor et al., 2013a) was obtained from a single orange filament cleaned of epibionts.

In the course of analyzing this genome, numerous short direct repeats of the heptanucleotide TAACTGA were noticed, particularly in intergenic regions directly upstream of translational start codons. The genomes of the marine *Beggiatoaceae Cand*. “Thiomargarita nelsonii” and *Thioploca ingrica*, and *Thiocystis violascens* (*Chromatiaceae*)—but not the freshwater *Beggiatoa alba*—also feature these repeats to varying degrees. Database searches further found TAACTGA direct repeats in some Cyanobacteria and a few Bacteroidetes, consistent with earlier evidence (MacGregor et al., 2013c) for genetic exchange between these groups and the *Beggiatoaceae*.

Tandem direct repeats of short nucleotide sequences have a very sporadic distribution in bacteria. In a comprehensive study, Mrázek et al. (2007) examined the distribution of what were termed long simple sequence repeats (LSSR) in prokaryotic genome sequences available at the time (2007). Repeat units of 1–11 nt were considered, and “long” was defined as series of repeats longer than statistically expected in a given genome. Species rich in LSSRs could be divided into those with repeat units primarily 1–4 or 5–11 nt long. They were phylogenetically scattered: for example, the 10 genomes identified with the most 5–11 nt repeats included four Betaproteobacteria (all *Burkholderia* spp.), two Cyanobacteria, three Actinobacteria, and one Gammaproteobacterium (*Xanthomonas campestris* ATCC 33913). Heptanucleotide repeats were the most abundant category in most genomes; it was proposed interaction of these with DNA polymerase might favor slippage and therefore duplications or deletions, and that 7 nt might be the length of sequence interacting with the polymerase. It was also noted that repeat units whose lengths are multiples of three were the most likely to be found within coding regions, presumably because series of them can be expanded and contracted without truncating a protein as long as they do not generate stop codons.

The same group went on to examine the genome-wide distribution of LSSR in several host-adapted pathogenic bacteria (Guo and Mrázek, 2008). Such repeats have been proposed and in some cases demonstrated to be involved in phase variation via slippage during DNA replication, turning on or off expression of virulence functions at either the transcriptional or the translational level. Some LSSR were in fact associated with antigenicity functions, such as envelope biogenesis genes, but COG classifications including these were not significantly overrepresented among the very diverse repeat-associated genes.

The genome-wide distribution of SSR (here abbreviating “simple satellite repeats”) in *Escherichia coli* has also been examined (Gur-Arie et al., 2000), considering only 1–6 nt units. For tetranucleotides, the longest unit reported in this regard, 78.9% of repeats were found in coding regions—very nearly the same proportion of the whole genome that is coding (79.5%). The repeats in intergenic regions did not show any particular concentration near translational start sites.

The two experimentally studied examples of bacterial tandem repeats between a promoter and a start codon are both upstream of surface proteins involved in phase variation in the respiratory pathogen *Moraxella catarrhalis*. A tract of either 9 or 10 G residues occurs 30 nt upstream of the translational start for the UspA1 gene (Lafontaine et al., 2001), which allows adhesion of the bacterium to human epithelial cells. Nine-residue G tracts were associated with high expression and 10-residue tracts with low expression. The tetranucleotide AGAT is found in strain-dependent copy numbers (from 6 to 23) in the 5′ untranslated regions of mRNAs for UspA2 (Attia and Hansen, 2006), a surface protein conferring resistance to human serum. Mutational studies in one strain found highest UspA2 expression with 18 copies.

This study describes the distribution of TAACTGA heptamer repeats in the BOGUAY genome, and the limited number of other species in which they have been found. Possible roles in translational regulation and genome rearrangement will be considered, depending on the length and position of the different repeat arrays. A possible alternate or derived RNA polymerase beta prime subunit gene identified in the Orange Guaymas “Maribeggiatoa” and *Thioploca ingrica* genomes is also discussed.

## Materials and methods

An orange tuft retrieved from core 4489-10 from RV *Atlantis*/HOV *Alvin* cruise AT15-40 (13 December 2008) at the UNC Gradient Mat site in Guaymas Basin, Gulf of California, Mexico (latitude 27° 0.450300′ N, longitude 111° 24.532320′ W, depth 2001 m) was cleaned of epibionts; its DNA amplified, tested for genetic purity, sequenced, assembled, and annotated; and the genome sequence checked for completeness, as previously described (MacGregor et al., 2013a,c). A total of 99.3% of the sequence was assembled into 822 contigs, suggesting good coverage was achieved. 4.7 Mb of sequence was recovered, with 80% of it forming large (≥15 kb) contigs. Throughout this paper, the genome is referred to as BOGUAY (from “*Beggiatoa* orange Guaymas”) and annotated sequences are referred to by 5-digit contig and 4-digit open reading frame (ORF) numbers (e.g., 00024_0691) or by ORF number alone (e.g., BOGUAY_0691). Additional sequence analysis was carried out using a combination of the JCVI-supplied annotation, the IMG/ER (Markowitz et al., 2009) and RAST (Aziz et al., 2008) platforms, and BLASTN, BLASTX, and BLASTP and PSIBLAST searches of the GenBank nr databases. Nucleic acid and amino acid sequence alignments were performed in MEGA5 (Tamura et al., 2011) using MUSCLE (Edgar, 2004) and small adjustments made manually. For identification of other TAACTGA-containing genomes, the GenBank nr database was searched with seven direct repeats of the TAACTGA sequence, using the default “short query” settings. For each strain with a sequence identified by this search, the genome sequence was searched for all TAACTGA direct repeats (in both orientations). RNA structure predictions are the first results from a minimum free energy calculation using the default settings of the MaxExpect algorithm from the RNAstructure Web Server (http://rna.urmc.rochester.edu/RNAstructureWeb/, Reuter and Mathews, 2010). Translations were done via the ExPASy portal of the Swiss Institute of Bioinformatics (Artimo et al., 2012). Protein domains were identified in CDD (Marchler-Bauer et al., 2011).

## Results and discussion

### Overview of sequenced *Beggiatoaceae*

The *Beggiatoaceae* family of giant sulfur bacteria includes species with a range of morphologies and habitats, very few of which have as yet been cultivated. Their classification is still in progress (Salman et al., 2011, 2013), but it is clear that many strains formerly designated *Beggiatoa* should be reclassified. Genomic sequence data are currently available for a small but diverse selection of these: complete or near-complete genome sequences for *B. alba* B18LD (Lucas et al. unpublished), *Thioploca ingrica* (Kojima et al., 2015), and Orange Guaymas “Maribeggiatoa” (MacGregor et al., 2013a,b,c); a partial sequence for *Cand*. “Thiomargarita nelsonii” (Mußmann et al., unpublished); and very partial sequences for two single filaments from the Baltic Sea, designated *Cand*. “Isobeggiatoa” PS and SS (Mussmann et al., 2007). By 16S rRNA gene sequence analysis, *B. alba* is in a separate clade from the rest of these (Salman et al., 2013).

### Abundance and distribution of TAACTGA repeats in the BOGUAY and other *Beggiatoaceae* genomes

**Figure 1:**
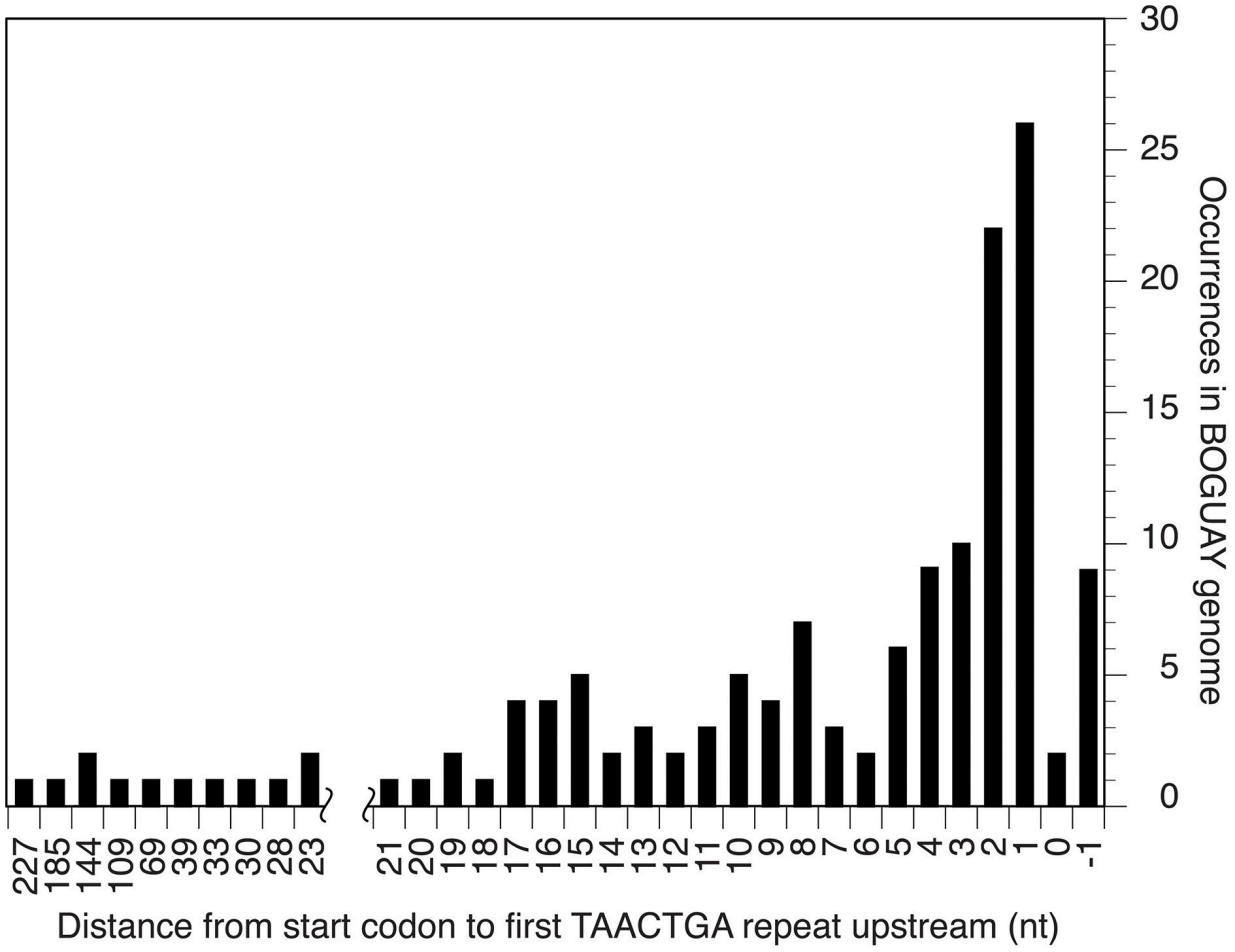
**Distance between start codon and first repeat for BOGUAY intergenic TAACTGA repeats**.

**Figure 2:**
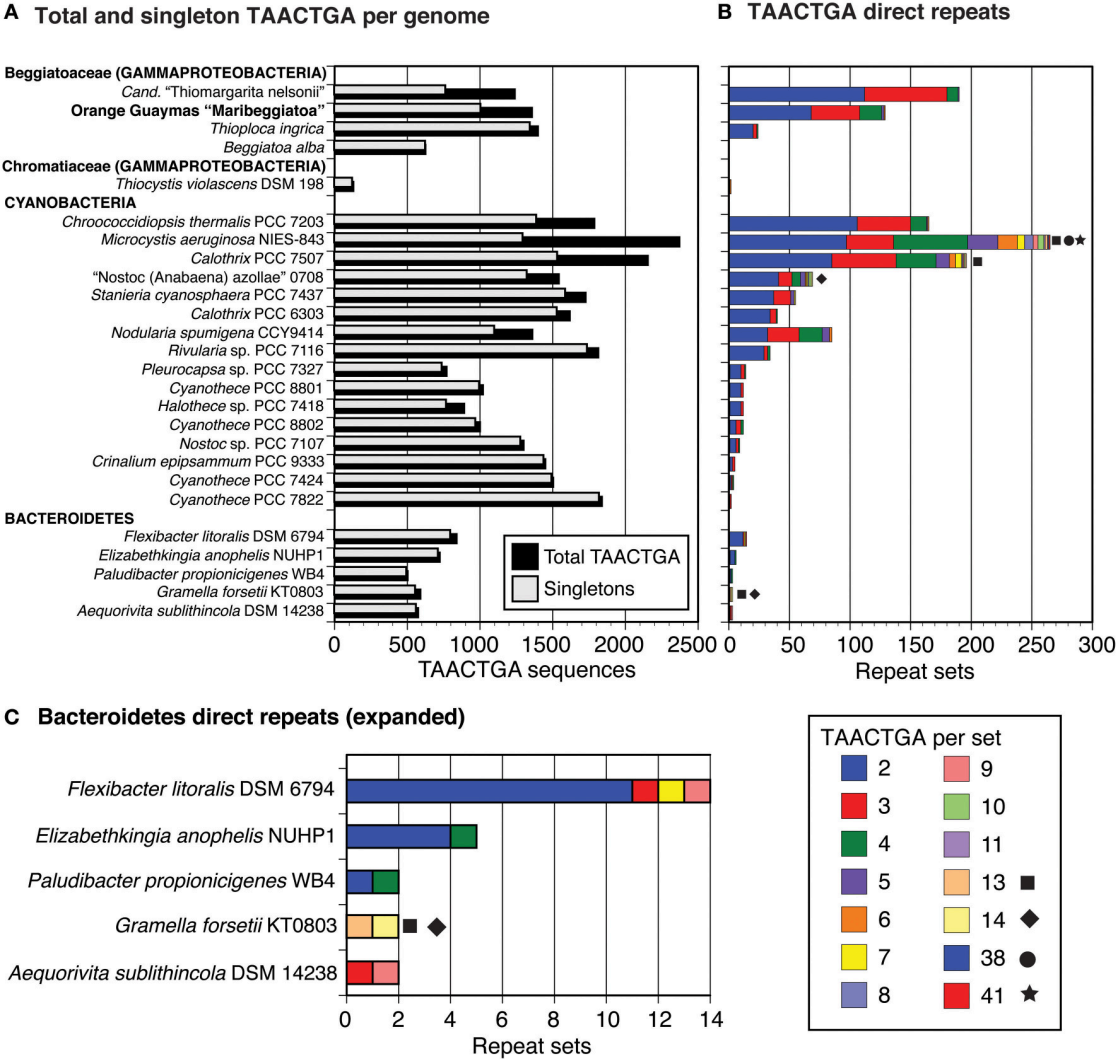
**Number and length of TAACTGA repeat sets in different species**. The GenBank nr database was searched with seven direct repeats of the TAACTGA sequence, using the default “short query” settings. For each strain with a sequence identified by this search, the genome sequence was searched for all TAACTGA direct repeats (in both orientations), and these were classified by the number of repeats they contain. The strains were sorted in order of number of two-repeat copies within each phylogenetic group. *Beggiatoa alba* contains no sets of repeats, but was included to present a complete set of available *Beggiatoaceae* genomes. **(A)** Total and singleton repeats in all species. **(B)** Repeat sets, classified by length. Especially long sets are highlighted by symbols. **(C)** Expanded view for Bacteroidetes repeat sets.

The Orange Guaymas “Maribeggiatoa” (BOGUAY) genome, with ~5330 annotated genes, contains some 169 sets of direct TAACTGA repeats and one indirect repeat, with between two and six copies per set (Table 1). Thirty-six of the sets are split by one or two different but related 7 bp sequences. Their distribution is not random: most are in a “forward” orientation upstream of a putative start codon, with the largest single category ending 1 nt upstream (Figure 1). All but 25 sets are completely intergenic. Of the rest, 14 overlap the end of an upstream ORF, with 13 in forward orientation to a downstream ORF; 10 are interior to ORFs in reverse orientation (Supplemental Table 1); and one is an inverted repeat near the end of an ORF, with the repeat units separated by one base pair (Table 2). There are an additional 819 singletons, whose distribution was not examined, for a total of 1357. TAACTGA repeats are also found in the “Isobeggiatoa” sp. PS and SS genomes, but these are too incomplete for thorough comparison. Of other sequenced *Beggiatoaceae*, Cand. “Thiomargarita nelsonii” has a similar number of repeats, and a higher proportion of doublets and triplets, but fewer longer sets; *T. ingrica* has a similar number of TAACTGA copies, but very few as direct repeats; and *B. alba* has less than half as many total copies and no direct repeats (Figures 2A,B, Supplemental Table 2).

**Table 1:** **Orientation of TAACTGA repeats in the BOGUAY genome**.

**Repeats**	**Orientation**															**Total**
	**Toward start codon, no RBS**				**Toward start codon, with RBS**			**Toward stop codon**				**Contig end**	**Other**			
	**Intergenic**	**Overlap**	**In ORF**	**Total**	**Intergenic**	**Overlap**	**Total**	**Intergenic**	**Overlap**	**In ORF**	**Total**	**Total**	**Intergenic**	**In ORF**	**Total**	
2	40	3	3	46	10	1	11	8	–	–	8	2	–	1	1	68
2, split	15	–	–	15	1	–	1	1	–	–	1	–	1	–	1	18
3	27	4	1	32	2	1	3	1	1	1	3	2	–	–	–	40
3, split	11	1	1	13	–	–	–	–	–	–	–	–	–	1	1	14
4	15	3	1	19	–	–	–	–	–	–	–	–	–	–	–	19
4, split	4	–	–	4	–	–	–	–	–	–	–	2	–	–	–	6
5	2	–	–	2	–	–	–	–	–	–	–	–	–	–	–	2
5, split	–	–	–	–	–	–	–	–	–	–	–	–	–	1	1	1
6	1	–	–	1	–	–	–	–	–	–	–	–	–	–	–	1
Inverted repeat	–	–	–	–	–	–	–	–	–	–	–	–	–	1	1	1
Total	115	11	6	132	13	2	15	10	1	1	12	6	1	4	5	170

“*Split” sets have a different but related 7-mer between two TAACTGA sequences*.

**Table 2:**
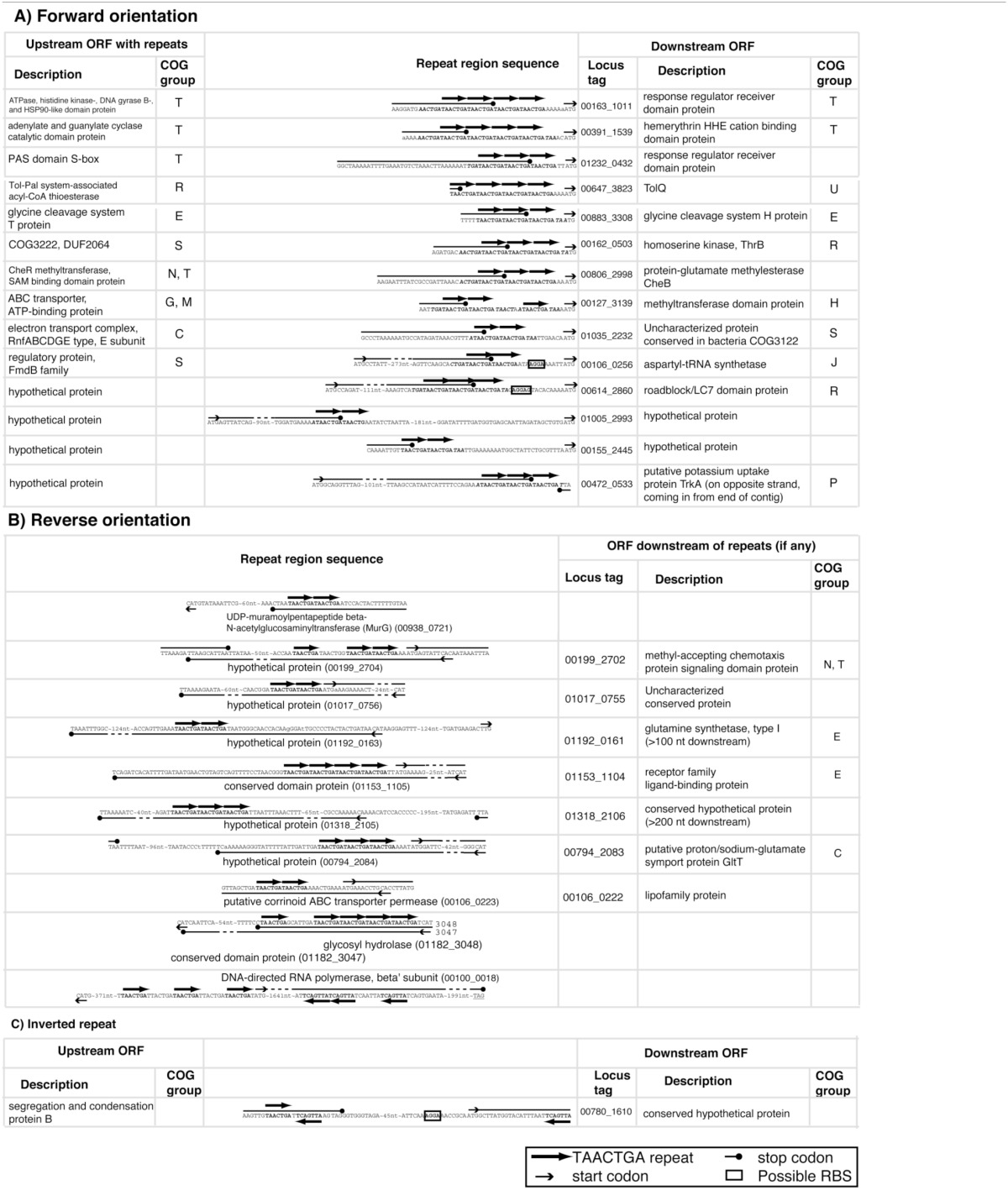
**TAACTGA repeats within or overlapping BOGUAY ORFs**.

### Direct repeats of sequences similar to TAACTGA are rare in the BOGUAY genome

A survey of the BOGUAY genome for heptamers with a single-base difference to TAACTGA (Table 3) showed that while some of these are in similar or greater abundance than TAACTGA as singletons, the maximum number of doublets for any of them was six, and only two had any longer sets of direct repeats (one of four units, one of six). Several scrambled versions of TAACTGA were also searched; all are at lower to considerably lower abundance as singletons, and none is found as even a single direct repeat. Factors such as coding potential likely influence the distribution of each of these, and some permutations may be selected against as interfering with whatever function(s) TAACTGA repeats may have, but TAACTGA does appear to be a favored sequence.

**Table 3:** **TAACTGA-like sequences in the BOGUAY genome**.

**DNA sequence (forward)**	**Total and direct-repeat occurrences in BOGUAY genome**						**Predicted RNA minimum free energy structure for six direct repeats**				**Amino acid repeat unit**	
	**Total copies**	**Repeats in set**					**Forward**		**Reverse complement**		**Forward**	**Reverse complement**
		**2**	**3**	**4**	**5**	**6**	**Type**	**kcal mol**^−1^	**Type**	**kcal mol**^−1^		
**TAACTGA AND SINGLE-BASE MUTATIONS**												
TAA***T***TGA	1908	6					Stem-loop	−4.3	One pair	3.5	LIIDN–	SIINYQL
TAA***A***TGA	1416	1					One pair	3.6	One pair	3.5	MINDK–	SFIIYHL
TAACTGA	1357	68	40	19	1	1	One pair	3.9	One pair	3.9	LITDN–	SVISYQL
***A***AACTGA	1354	4					One pair	3.9	One pair	3.9	KLKTEN-	SVFSFQF
TAACT***C***A	1206						One pair	3.9	Stem-loop	−10.2	LITHNS-	VMSYEL-
TAACT***T***A	1018						Stem-loop	−6.1	Stem-loop	−9.7	LITYNL-	VISYKL-
TA***T***CTGA	943						Stem-loop	−14.6	Stem-loop	−13.4	YLISDI-	SDIRYQI
***C***AACTGA	829	3					One pair	3.1	Stem-loop	−6.3	QLTTDN-	SVVSCQL
T***T***ACTGA	810					1	Stem-loop	−4.4	One pair	3.9	LLITDY-	SVISNQ-
TAACT***A***A	786	4		1			One pair	3.9	Stem-loop	−4.3	LITNN–	LLVISY-
T***C***ACTGA	786	1					Stem-loop	−15.1	Stem-loop	−21.2	SLITDH-	SVISDQ-
T***G***ACTGA	778						One pair	3.9	One pair	3.9	LMTDD–	SVISHQS
TAAC***G***GA	742	1					Stem-loop	−4.6	One pair	3.4	RITDNG-	SVIRYPL
TA***C***CTGA	701						One pair	3.9	Stem-loop	−11.0	YLIPDT-	SGIRYQV
TAA***G***TGA	632						One pair	3.2	One pair	3.9	VISDK–	SLITYHL
TAACTG***T***	625	1					Stem-loop	−12.8	Stem-loop	−11.8	LLTVNC-	QLTVNS-
***G***AACTGA	606						Stem-loop	−4.1	Stem-loop	−2.2	ELRTEN-	SVLSSQF
TA***G***CTGA	598						Stem-loop	−18.4	Stem-loop	−10.4	LIADS–	SAISYQL
TAAC***A***GA	544						One pair	3.9	One pair	3.8	QITDNR-	SVICYLL
TAACTG***G***	476						Stem-loop	−11.8	Stem-loop	−14.4	LVTGNW-	PVTSYQL
TAAC***C***GA	470	1					One pair	3.4	Stem-loop	−12.8	PITDNR-	SVIGYRL
TAACTG***C***	351						Stem-loop	−4.0	Stem-loop	−11.8	LLTANC-	QLAVSS-
**SHUFFLED TAACTGA (SELECTION)**												
ATATCAG	1030						Stem-loop	−13.4	Stem-loop	−14.0	ISDIRYQ	YLISDI-
ATAATCG	867						Stem-loop	−14.0	Stem-loop	−15.4	SIIDNR-	RLSIIDY
CTAAGTA	322						Stem-loop	−13.8	Stem-loop	−14.5	VLSTKY-	YLVLST-
TCGAATA	319						Stem-loop	−9.4	Stem-loop	−11.0	SNIEYRI	YSIFDIR
TAACTAG	160						Stem-loop	−16.6	Stem-loop	−15.6	LVTSN–	LLVTSY-

*DNA sequences are arranged by number of occurrences. The TAACTGA sequence itself is outlined. Single-base differences to it are in bold italics. For each DNA sequence, an RNA structure was predicted for six direct repeats. Amino acid sequences were predicted for 7 direct repeats, but only a single repeat unit is shown. Shaded boxes indicate amino acid sequences containing stop codons. RNA structure predictions are the first results from a minimum free energy calculation using the default settings of the MaxExpect algorithm from the RNAstructure Web Server [http://rna.urmc.rochester.edu/RNAstructureWeb/, (Reuter and Mathews, 2010)]. Translations were done via the ExPASy portal of the Swiss Institute of Bioinformatics (Artimo et al., 2012)*.

### Predicted characteristics of RNA and amino acid sequences that might be produced from TAACTGA repeats

If the BOGUAY TAACTGA repeats have common function(s), these could be at the DNA, RNA, or in a few cases protein level. At the DNA level, repeat sequences can serve as recombinational and mutational hot spots (reviewed in Lovett, 2004; Zhou et al., 2014), or as binding sites for regulatory proteins. They could conceivably also mark the site of transposon excisions; some transposon insertions can generate 7 nt direct repeats (Sallam et al., 2006), although in the studied cases they seem usually to resolve to singletons upon excision (Foster et al., 1981).

At the RNA level, the repeats may again be protein-binding sites (or interrupt existing ones), and/or impart secondary structure. As direct repeats in up to six copies, however, TAACTGA is not predicted to generate any particular RNA secondary structure in either orientation (Table 3), unless by interaction with surrounding sequences.

At the protein level, translation of TAACTGA and its reverse complement (TCAGTTA) reveals what is probably a major factor controlling genomic distribution of these sequences. In the “forward” orientation, translation of TAACTGA repeats yields the repeating amino acid sequence LITDN–, where dashes represent stop codons. These can therefore overlap the end of coding sequences by no more than 18 nt, or two full repeats plus four nucleotides. If repeats are carried by mobile elements, their introduction into coding sequences in forward orientation will terminate the gene, and usually be deleterious. In some locations it might be tolerated however, for example between the subunits of modular proteins, or at the beginning or end of a protein. Possible examples will be discussed below.

Translation of repeats in the “reverse” orientation yields the repeating sequence LSVISYQ. At first glance, this suggests a leucine zipper dimerization domain (reviewed in Parry et al., 2008), with nonpolar residues in the first (L) and fourth (I) positions, but there are no charged amino acids for interactions on the other face of the predicted helix, and the nonpolar third position (V) is unusual. According to the algorithm of Bornberg-Bauer et al. (1998), this sequence does not have the requisite leucine zipper coiled-coil structure even when 20 or more amino acid repeats are included. *Ab initio* structure predictions (Xu and Zhang, 2012) for a peptide composed of seven LSVISYQ repeats (and several variants) suggest a structure dominated by antiparallel beta sheets (not shown), but structure in a real protein would depend on the number of repeats and on interactions with the rest of the protein.

Compared to other similar heptamers, TAACTGA has no obvious special features (Table 3): several have similar genomic abundances, many yield apparently similar local RNA conformations, a majority can be translated in “reverse” orientation, and all single-base mutants yield one or more stop codons in “forward” orientation. None of these properties shows a strong correlation with chromosomal abundance, or with occurrence as direct repeats. Assuming all relevant properties have been considered, this is consistent with TAACTGA repeats arising in one lineage and being horizontally transferred to others. The alternatives that this particular sequence became repeated independently in multiple isolated lineages, or was preserved as such in only a few, seem less likely.

### Abundance and distribution of TAACTGA repeats in the Cyanobacteria and Bacteroidetes

A GenBank search for TAACTGA direct repeats found a very limited phylogenetic distribution (Figure 2). Outside of the *Beggiatoaceae*, considering only complete or near-complete genomes, TAACTGA repeats were identified in one other sulfur-oxidizing Gammaproteobacterium (*Thiocystis violascens* DSM 198), 15 Cyanobacteria, and 5 Bacteroidetes. This distribution is similar to that previously noted for the *fdxN* element excision-controlling factor proteins XisH and XisI (MacGregor et al., 2013c). An updated (May 2015) database search found that at least one of these was annotated in all cyanobacterial genomes with TAACTGA repeats except *Stanieria cyanosphaera* PCC 7437, but not in the Bacteroidetes represented (although they are found in some other genera in this group) and not in *T. ingricans* or *T. violascens* (Supplemental Table 7). The hypothetical protein BOGUAY_0693, which has 29 close matches in the BOGUAY genome, has matches in some but not all of the same cyanobacteria, the other *Beggiatoaceae*, and *Flexibacter litoralis*, but not in the remaining Bacteroidetes or *T. violascens* (Supplemental Table 7). Whether or not a common transfer mechanism is involved, this is consistent with a history of genetic exchange among some Cyanobacteria and *Beggiatoaceae*.

As in the *Beggiatoaceae*, there is no necessary correlation between number of singletons and number of repeats (Figure 2, Supplemental Table 2); for example, *Cyanothece* PCC 7424 has more singleton and nearly as many total copies as “Nostoc azollae” 0708, but 3 vs. 69 sets of repeats. There are no obvious morphologies, metabolic types, or habitats common to all the species found: for example, *Microcystis aeruginosa* NIES-843 (NC7) is a colonial freshwater cyanobacterium isolated in Japan (Otsuka et al., 2000); *Elizabethkingia anophelis* NUHP1 is a Gram negative rod from a mosquito midgut collected in The Gambia (Kämpfer et al., 2011); and *Aequorivita sublithincola* DSM 14238 is an endolithic Gram negative bacterium found as rods or filaments, isolated from within a quartz rock in Antarctica (Bowman and Nichols, 2002). This complicates the argument just made for horizontal transfer; characterization of other heptamer repeats and additional genomic sequencing may clarify this issue.

#### Cyanobacteria

Among the Cyanobacteria, the sequenced genomes of the freshwater, bloom-forming *M. aeruginosa*, particularly strains NIES-843 (Kaneko et al., 2007) and PCC 7806 (Frangeul et al., 2008), have high proportions of repeated sequences. This has been proposed to be part of an evolutionary strategy relying on genome plasticity, with a comparatively high number of horizontally acquired genes and repeated genes and sequences (Humbert et al., 2013). These include a range of repeating heptamers, with TAACTGA repeats often mixed with others. A complete analysis was not carried out here, but a small random sample of the 265 sets of *M. aeruginosa* NIES-843 TAACTGA repeats suggests that they may play more or different roles than in BOGUAY. Of 24 sets of repeats mapped in detail (Supplemental Table 3), 22 were intergenic and two in “reverse” orientation within ORFs encoding small hypothetical proteins. Of the intergenic sets, just six were in “forward” orientation relative to a downstream start codon, and at a range of distances (from 1 to 214 nt). Eight sets were in reverse origin relative to a start codon and eight were between stop codons. All of the latter are in the same orientation on the chromosome; it would be interesting to see whether this pattern holds throughout the genome. If this is a representative sample, it is a clear contrast to the BOGUAY genome, where most sets of repeats are intergenic and in “forward” orientation to a relatively nearby start codon. The chromosomal arrangement is not known because the genome is not closed.

Repeat distributions in four *Cyanothece* strains with relatively few TAACTGA copies were also compared (Supplemental Table 4). *Cyanothece* PCC 8801 and 8802 are very similar, with nine sets of repeats in matching positions in terms of flanking ORFs and only small intergenic sequence differences, mostly indels in 7 nt increments. Seven of these repeat sets are just upstream of a start codon, one just upstream of a putative Shine-Dalgarno (SD) sequence, and one in reverse orientation near the upstream ORF. PCC 8802 has an additional intergenic set relatively far upstream from a start codon; each strain has an intergenic plasmid-borne set, but between different ORFs; and PCC 8801 has one set in reverse orientation internal to an ORF. In PCC 7424, there are only three sets of repeats, none in positions matching the other two strains. All are intergenic and in “forward” orientation, at varying distances from the nearest start codon. The closest relatives of the flanking ORFs are all from strain PCC 7822, including those flanking its only set of repeats. Overall, whether TAACTGA and related repeats derive from a common cyanobacterial ancestor or are transmitted by some mobile element, they appear to have followed strain-specific paths here as in other lineages.

#### Bacteroidetes

The distribution of TAACTGA repeats in the Bacteroidetes (Figure 2C) suggests they could also have more than one role in this group. *F. litoralis* DSM 6794 is similar to BOGUAY, on a more limited scale. Of 14 repeat sets, 12 are intergenic and in the “forward” orientation relative to a start codon between 1 and 43 nt downstream (Supplemental Table 5). One set of seven repeats is located immediately downstream of a stop codon, in reverse orientation, and a set of two is located within a putative PurC (SAICAR synthase) gene, near its end. In *Paludibacter propionicigenes* WB4 there are just two sets of direct repeats, one close to a start codon and the other toward the center of a long intergenic region (Supplemental Table 5).

The remaining three Bacteroidetes strains have different distributions. *Gramella forsetii* KT0803 and *A. sublithincola* DSM 14238 have only two sets of TAACTGA direct repeats each, but three of these are quite long (Figure 2C). All are intergenic and in “forward” orientation relative to the downstream ORF, but only one is immediately upstream of a start codon, and the intergenic regions contain other heptamer direct repeats as well (Supplemental Figure 3). For both *A. sublithincola* sets and one of the *G. forsetii* ones, the closest matches to the upstream and downstream ORFs are found in the same close relative (*A. capsosiphonis* DSM 23843 and *G. echinicola* DSM 19838, respectively), which have shorter intergenic regions without obvious sets of repeats, although the immediate gene neighborhoods appear the same (Supplemental Figure 3A). In the second *G. forsetii* example (Supplemental Figure 3B), at least the downstream ORF may have been acquired by horizontal transfer. The closest match to the upstream ORF is from the Bacteroidetes strain *Gillisia limnaea* DSM 15749, which has a similar local gene neighborhood, except that instead of a homolog of the downstream ORF there is a short hypothetical protein encoded on the opposite strand. No sets of direct repeats are evident in this intergenic region. Downstream, the closest match to the *G. forsetii* ORF is from *Bacillus azotoformans* LMG9581, which has no other apparent local similarity to *G. forsetii*. A phylogenetic reconstruction for this ORF and a comparison of intergenic regions in other *Gramella, Gillisia*, and *Bacillus* strains would be needed to propose a history for this small region, but the pattern so far suggests a role in gene rearrangement for these intergenic repeats.

*E. anophelis* NUHP1 has sets of TAACTGA repeats between only three pairs of ORFs, which are not very long (four sets of two, one set of four), but in two cases they are part of nearly identical intergenic regions containing larger assemblages of heptamer repeats and flanked by ORFs encoding putative proteins with stretches of high identity (Supplemental Table 6). Comparisons with closest neighbors (all *Elizabethkingia* strains) were difficult because the contigs identified often end partway through the repeat region, likely because of assembly difficulties. The third repeat set is a single pair, found toward the center of a relatively long (295 bp) intergenic region with no other obvious repeats.

### Canonical ribosome binding sites are rare in repeat-containing BOGUAY intergenic regions

The TAACTGA repeats in the BOGUAY genome are generally positioned close to start codons (Figure 1), overlapping the expected ribosome binding site. The Shine-Dalgarno (SD) sequence predicted from the 16S rRNA genes of BOGUAY and other sequenced *Beggiatoaceae* is the same as that of *E. coli* (AGGAGGU). With only one G residue per heptamer in either orientation, the repeat sequence itself has little SD character, so most of the ORFs downstream of them have no obvious ribosome binding site. For an overview of the genome, any four consecutive bases from the AGGAGGU sequence ending 4–13 nt upstream of a start codon was considered an SD, recognizing that this may lead to over- or undercounting. The number of such sequences was estimated at 1346 (Supplemental Table 8), accounting for 25% of the 5272 predicted protein-coding genes. This is toward the low side for bacteria overall, but by no means unmatched (Ma et al., 2002). Of intergenic regions with repeats, just 15 (~10%) also include SD sequences (Table 4), with the repeats ending between 2 and 25 bp upstream of them.

**Table 4:**
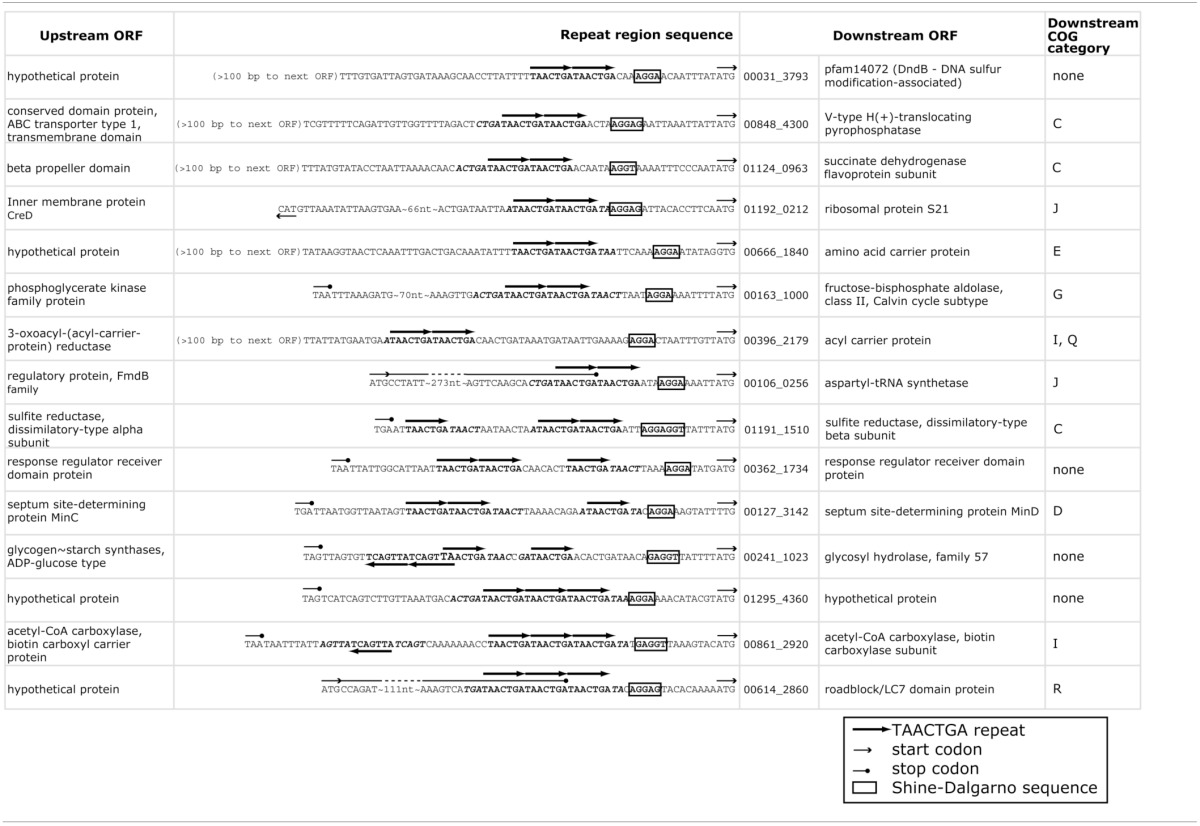
**BOGUAY ORFs preceded by both Shine-Dalgarno sequences and TAACTGA repeats**.

*Shine-Dalgarno sequences are defined, here and throughout, as having 4 or more consecutive matches to the consensus sequence AGGAGGT. Entries are organized to highlight similar arrangements of SDs and repeats*.

### Functional classification of BOGUAY ORFs downstream of TAACTGA repeats

The COG (Clusters of Orthologous Groups; Tatusov et al., 1997) classifications of ORFs with and without upstream repeats were compared (Table 5). Categories F, D, Q, E, and J were particularly overrepresented among those with repeats, while only category A was as strongly underrepresented. Note however that 63% of all ORFs and 29% of those with repeats have not been classified at all, and some 8% more of each are in categories R (general function prediction only) and S (function unknown). No clear picture of a possible transcriptional or translational regulatory role for TAACTGA repeats is apparent at this level, particularly since it is not known whether regulation is positive or negative. Several concentrations of repeat sequences will be considered in more detail below.

**Table 5:** **COG classification of BOGUAY ORFs downstream of TAACTGA repeats compared to whole genome**.

**COG category**		**Number of ORFs downstream of repeats**	**% of ORFs downstream of repeats**	**% of all ORFs**	**Fold difference (%repeats/%total)**
**OVERREPRESENTED DOWNSTREAM OF TAACTGA REPEATS**					
F	Nucleotide metabolism and transport	6.5	4.55	0.82	5.6
D	Cell cycle control and mitosis	3	2.60	0.62	4.2
Q	Secondary metabolites	1.8	1.95	0.47	4.1
E	Amino acid metabolism and transport	12.5	9.09	2.36	3.9
J	Translation	13	8.44	2.39	3.5
H	Coenzyme metabolism	7.5	5.19	1.78	2.9
I	Lipid metabolism	2.8	1.95	0.80	2.4
O	Post-translational modification, protein turnover, chaperone functions	7	4.55	1.94	2.3
G	Carbohydrate metabolism and transport	4	2.60	1.16	2.2
M	Cell wall/membrane/envelope biogenesis	7	5.84	3.12	1.9
T	Signal transduction	7	4.55	2.48	1.8
C	Energy production and conversion	6.5	4.55	2.85	1.6
V	Defense mechanisms	1	0.65	0.45	1.4
K	Transcription	1.5	1.30	1.23	1.1
**UNDERREPRESENTED**					
U	Intracellular trafficking and secretion	2	1.30	1.49	0.9
L	Replication and repair	1.5	1.30	1.69	0.8
N	Cell motility	0.5	0.65	1.16	0.6
P	Inorganic ion transport and metabolism	1	0.65	1.83	0.4
A	RNA processing and modification	0	0.00	0.04	0.0
**UNCATEGORIZED**					
R	General functional prediction only	6.8	4.55	4.28	1.1
S	Function unknown	8	4.55	3.91	1.2
None assigned		46	29.22	63.16	0.5
TOTAL		147	100.00	100.00	

*Fractional occurrences were used for ORFs assigned to more than one category*.

### TAACTGA repeats within open reading frames

While most of the TAACTGA repeats in the BOGUAY genome are intergenic, suggesting a regulatory role, there are exceptions. The coding regions of 25 putative BOGUAY proteins contain or overlap 24 sets of direct repeats, with one set found in overlapping ORFs (BOGUAY_3048 and _3047). In 13 of these, between one partial and two complete repeats overlap the stop codon of an upstream gene in “forward” orientation relative to a downstream gene (Table 2A); as mentioned above, forward repeats generate stop codons in all three reading frames, so these are necessarily at the end of ORFs. In only two of these was a recognizable SD sequence found between the end of the repeats and the start codon of the downstream ORF. In three more ORFs, sets of repeats were found within or overlapping one end or the other of the putative coding sequence, but not directly upstream of another (Table 2B). One example was also found of an indirect repeat near the end of an ORF, with one base pair separating the two copies (Table 2C).

The 11 ORFs containing “reverse” repeats (Table 2B) have no apparent amino acid sequence similarity outside the repeat-encoded region (Supplemental Figure 1). Seven are short hypothetical or conserved-domain proteins with no assigned functions. One of these overlaps a putative glycosyl hydrolase (BOGUAY 01182_3048); the repeat-encoded amino acids in the latter are near the C-terminal end of the predicted protein, with little homology to otherwise close database relatives and outside the CDD-defined glycosyl hydrolase domain that includes most of the rest of the ORF (not shown). The repeat-encoded amino acids of two of the other ORFs with assigned functions are likewise outside regions of assigned function, either toward the very beginning (corrinoid ABC permease, BOGUAY 00106_0223) or very end (MurG, BOGUAY 00938_0721) of their respective amino acid sequences. The exception is BOGUAY 00100_0018, an ORF encoding a putative protein similar to an RNA polymerase beta prime subunit, discussed below.

If the repeats were or are mobile within the genome, their insertion within coding sequences seems to have been successful primarily at the periphery of at least the primary structure of proteins. For repeats in “forward” orientation, this is a necessary consequence of their sequence, which encodes stop codons in all three reading frames. “Reverse” repeats could in principle occur anywhere, but most insertions are likely deleterious. Those at the end of proteins, or perhaps splitting a protein into two new functional proteins, are probably more likely to become fixed.

Direct TAACTGA repeats are also found within hypothetical proteins in *Beggiatoa* sp. PS and some cyanobacteria, particularly *M. aeruginosa* strains. A BLASTP search of the GenBank protein database with 7, 14, or 21 LSVISYQ repeats yielded mostly predicted amino acid sequences annotated as hypothetical proteins. The shorter variant yielded the most perfect matches (Supplemental Table 9). The phylogenetic distribution of at least the top hits was quite restricted: 61 cyanobacterial sequences, of which 25 were from *M. aeruginosa* and 25 from *Moorea producens*; 17 Gammaproteobacterial sequences, of which 12 were from *Pseudoalteromonas* spp. and 2 from *Beggiatoaceae*; 9 from the Betaproteobacterium *Burkholderia pseudomallei*; 6 from Alphaproteobacteria, of which 4 were from *Ehrlichia ruminantium*; and one reportedly from a bird. Interestingly, one of these was annotated as an FdxN element excision controlling factor protein-like protein (BAG05441.1 from *M. aeruginosa* NIES-843). However, given the large number of these in the database, and the fact that it has no BLASTP matches from this group, this is suspected to be a misannotation. Similarly, the *B. pseudomallei* predicted protein (KGC53376) described as a putative 60S ribosomal protein L19 does not seem to actually belong to this group.

### TAACTGA repeats in putative ribosomal protein operons

**Figure 3:**
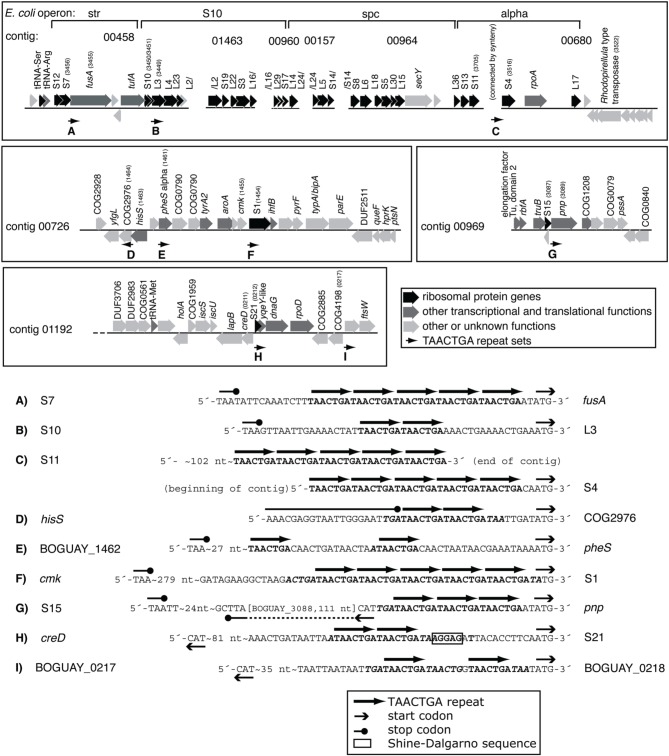
**TAACTGA repeats in and near putative BOGUAY ribosomal protein operons**. Repeats are found upstream of putative genes for **(A)** *fusA* (elongation factor G); **(B)** ribosomal protein L3; **(C)** ribosomal protein S4; **(D)** a COG2976 protein; **(E)** *pheS* (phenylalanine-tRNA ligase, alpha subunit); **(F)** ribosomal protein S1; **(G)** *pnp* (polynucleotide phosphorylase); **(H)** ribosomal protein S1; and **(I)** ORF BOGUAY_0218.

One COG category overrepresented among BOGUAY ORFs preceded by repeats is J (Translation) with 13 examples, including four upstream of putative genes for ribosomal proteins (S1, L3, S4, and S21; Figure 3) and five others within putative r-protein operons (*pnp, fusA*) or nearby (COG2976, *pheS*, BOGUAY_0218). Only one of these (S21, Figure 3H) also has a ribosome-binding site by the criteria used above. As described previously (MacGregor et al., 2013a), BOGUAY ribosomal protein genes are organized similarly to those in *E. coli* (see e.g., Fu et al., 2013 for an illustration) and many other bacteria. Where studied, these are transcribed as multigene operons, with translation generally regulated by a negative feedback loop involving one of the proteins encoded by the operon. Short noncoding RNAs transcribed within these operons may also play a role (Khayrullina et al., 2012). There is no experimental evidence regarding transcription of any BOGUAY genes, but it is worth noting that all TAACTGA repeats within BOGUAY r-protein operons are internal to the standard operons, suggesting a role in translational rather than transcriptional regulation. Insertion of a mobile element at such an internal site might also be favorable compared to insertion in a promoter region for these highly expressed operons, although given the essential role of ribosomes any insertion at all seems potentially disruptive.

This distribution has some overlap with the other *Beggiatoaceae* “T. nelsonii” and *T. ingrica*. In particular, all three species have TAACTGA repeats upstream of their putative S1 subunit genes (Supplemental Figure 2): BOGUAY has 5, beginning 1 nt upstream; “T. nelsonii” has three copies but with gaps between them, also beginning 1 nt upstream; and *T. ingrica* has 2 copies, beginning 21 nt upstream. The sequence of this gap is nearly identical (18 of 21 nt) to the *B. alba* sequence over this stretch; *B. alba* of course has no repeats. This shared sequence does not include a ribosome-binding site, by the definition used here, but does have an AGGG and an AGGGG run.

Three of the four putative BOGUAY r-protein genes preceded by repeats (S1, S4, and L3) are also among those with proposed extraribosomal functions in *E. coli* (reviewed in Aseev and Boni, 2011). During translation, S1 is involved in ribosome docking and in unfolding of structured mRNAs (Duval et al., 2013), interacting with AT-rich regions upstream of the SD sequence (if there is one), as well as with downstream sequences (Tzareva et al., 1994). In *E. coli*, S1 is required for translation of all mRNAs with leader sequences (reviewed in Hajnsdorf and Boni, 2012), while leaderless mRNAs can be translated by ribosomes lacking it (reviewed in Byrgazov et al., 2013). Like several other ribosomal proteins, it inhibits translation of its own operon: at least *in vitro*, free S1 competes with ribosome-bound S1 for mRNA binding upstream of the start codon (Boni et al., 2001). Again *in vitro*, it is reported to have a transcriptional role as well: *E. coli* S1 co-purifies with RNAP and stimulates transcriptional cycling (Sukhodolets et al., 2006).

The *E. coli* S4 ribosomal protein, in addition to negatively regulating translation of its own operon, is proposed to form part of transcriptional antitermination complexes that may also include L1, L3, and L4 (Torres et al., 2001), with S4 binding RNAP directly.

### Candidate repeat-binding proteins

The frequent position of the TAACTGA repeats upstream of and apparently replacing SD sequences, including five direct repeats directly upstream of the S1 gene (Figure 3), suggests that they might play a role in translation. Several categories of known translational regulatory proteins have properties that suggest them as candidates.

#### Ribosomal protein S1

Interaction with the S1 subunit is one possibility. S1 has a relatively weak and reversible association with the ribosome, and is added last in assembly (Subramanian and Vanduin, 1977). In *E. coli* and many other Gram negative bacteria, it is composed of six linked oligonucleotide/oligosaccharide binding (OB)-fold domains; where studied, the four C-terminal domains are RNA-binding, while the two N-terminal domains make protein-protein contacts with ribosomal, and other proteins (reviewed in Hajnsdorf and Boni, 2012). The BOGUAY S1 protein is predicted to have a typical Gram negative S1 structure (not shown).

The *E. coli* S1 gene itself (*rpsA*) lacks a strong SD sequence and does not require one for expression (Boni et al., 2001). The upstream region forms three hairpins, which contribute to its translational efficiency (Boni et al., 2001; Skorski et al., 2006). Different secondary structures can be predicted for the intergenic region upstream of the BOGUAY S1 gene, depending how much of this and the coding sequence are included in the calculation (not shown), but they have no obvious similarity to those in *E. coli*. Without experimental evidence, or knowledge of the transcriptional start site, they cannot be assigned a function. One argument against a TAACTGA-binding role for S1 is the reported non-specificity of S1 RNA recognition, limited to a preference for AT-rich sequences (reviewed in Aseev and Boni, 2011). TAACTGA repeats are somewhat AT rich, but do not produce long polypyrimidine tracts.

#### Cold shock proteins

As a second possibility, the cold shock proteins (CSPs; since shown to include proteins with other roles) are OB-fold proteins with a single S1-like domain that can bind single-stranded RNA or DNA. Intriguingly, X-ray crystallography (Sachs et al., 2012) and microarray binding (Morgan et al., 2007) studies of *Bacillus subtilis* CspB have shown that it can bind heptamer direct repeats (reviewed in Horn et al., 2007), with one protein per heptamer, although only weak sequence specificity (e.g., stronger binding to TTCTTTT than TTTTTT) has been demonstrated. During cold shock, CSPs bind both non-specifically to general RNA and specifically to the 5′ untranslated region of selected mRNAs; this selection has been proposed to rely more on secondary structure than primary sequence (Giuliodori et al., 2004), but limited work has been done on this question. It seems conceivable that some Csp-like proteins might bind in a sequence-specific manner.

There are several putative proteins with cold shock domains in the BOGUAY genome (Supplemental Table 10). Two include just a single cold shock domain, and are annotated as CspA and CspE; two have a downstream Excalibur calcium-binding domain; and one has a downstream DUF1264 domain. According to a CDD (Marchler-Bauer et al., 2011) search, the CSP-Excalibur architecture is found in 301 other proteins in the GenBank nr protein database, of which 298 are Proteobacterial; 256 of these are Gammaproteobacterial. Similarly, the CDS-DUF1264 architecture is found in 801 nr sequences, of which 766 are Proteobacterial and 614 Gammaproteobacterial. Cyanobacteria were the next largest group, but with just 13 examples. It is not uncommon for a single Gammaproteobacterial genome to encode more than one CSP domain protein (not shown).

PSORTb 3.0 (Yu et al., 2010) predicts the putative BOGUAY CspA and CspE to be cytoplasmic, by similarity to known proteins (Supplemental Table 10). The CSP-DUF1264 protein is predicted to possess four internal helices and be a cytoplasmic membrane protein, making it an unlikely translational regulatory protein. No prediction could be made for the two CSP-Excalibur putative proteins (while the name stands for “extracellular calcium-binding region,” this is due to the proteins the domain was originally identified in Rigden et al. (2003); other proteins containing it may or may not be extracellular). At least two (the putative CspA and CspE) and possibly four of these CSP-like proteins are therefore candidates for TAACTGA binding. While so-called cold-shock domain proteins need not respond to temperature, temperature is likely an important environmental clue in the Guaymas Basin microbial mats, signaling the intensity of the hydrothermal flow that supplies sulfide to the sulfide-oxidizing BOGUAY strain and its relatives.

#### CsrA-like proteins

As a third possibility, CsrA (*E. coli* carbon storage regulatory protein) and related proteins bind to single-stranded RNA, in some cases inhibiting translation by competing with ribosomes for binding to Shine-Dalgarno sequences. They play a role in processes including motility, biofilm formation, quorum sensing, and virulence in a wide range of bacteria (reviewed in Romeo et al., 2013; Van Assche et al., 2015). The BOGUAY genome contains a *csrA* candidate (BOGUAY 00153_2343) with a strong possible SD site (AGGAG, 7 nt from the start codon), consistent with the autoregulation often found for these genes (Romeo et al., 2013). However, the known RNA binding sites for CsrA proteins, whether on target RNAs or on regulatory small RNAs, are centered on SD-like GGA motifs with more than 7 nt spacing (reviewed in Duss et al., 2014). These are not found in TAACTGA repeats in either orientation, making these unlikely to be recognized by a canonical CsrA.

### Possible secondary or repurposed RNA polymerase beta prime subunits in BOGUAY and *Thioploca ingrica*

The BOGUAY genome encodes two putative RNAP β′ subunits (MacGregor et al., 2013c), an unusual feature also found in the recently sequenced *T. ingrica* genome, but not in *B. alba*. The partial “Isobeggiatoa” PS sequence includes only one. The *Cand*. “Thiomargarita nelsonii” genome is annotated with two (OT06_22820, OT06_51635), each on short contigs with no surrounding ORFs, but their sequences are identical except that OT06_51635 is missing 214 C-terminal amino acids where its contig ends; if this is in fact a duplication, it would seem to be a fairly recent one. Of 100 top BLASTP hits to BOGUAY 00100_0018, the alternate beta prime gene, only one intraspecific pair of beta prime genes was found. *Nitrosococcus watsonii* C-113 has an apparent tandem duplication of its beta (Nwat_2177, Nwat_2165) and beta prime (Nwat_2176, Nwat_2164) genes along with surrounding ribosomal protein and other translation-related genes. The two putative beta prime subunit genes are 100% identical at the nucleotide level; again, if this is a duplication, it appears recent.

**Figure 4:**
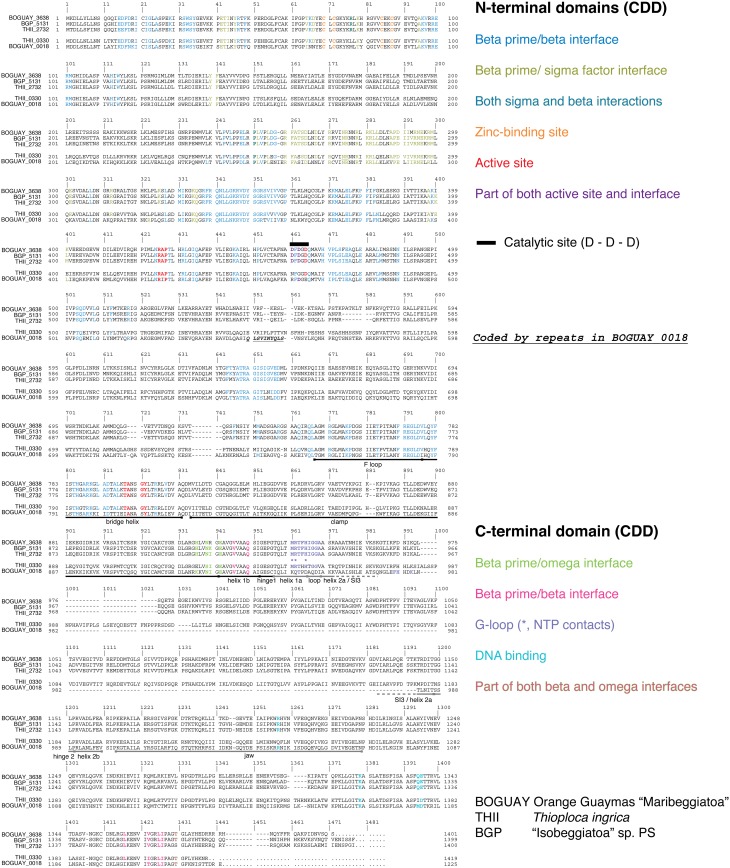
**Alignment of RNA polymerase beta prime and beta-prime like sequences from the BOGUAY, “Isobeggiatoa” PS, and ***Thioploca ingrica*** genomes**. Sequences were aligned in MEGA5.2.2 (Tamura et al., 2011) using Muscle (Edgar, 2004). Trigger loop and SI3 annotation are after Windgassen et al. (2014), F loop and bridge loop annotation after Miropolskaya et al. (2014), jaw annotation after Opalka et al. (2010), and clamp annotation after Davis et al. (2007). Other putative domains were identified in CDD (Conserved Domain Database; Marchler-Bauer et al., 2011). Active-site and G-loop regions are boxed, and details of these shown to the right of the complete alignment.

BOGUAY and *T. ingrica*, by contrast, each have two different beta prime-like genes (Figure 4). One of these (BOGUAY_3638, THII_2732) appears to include all the expected catalytic and subunit-interaction sites of a bacterial beta prime subunit, and is very similar to the single “Isobeggiatoa” PS sequence (BGP_5131). The other (BOGUAY_0018, THII_0330) has the N-terminal subunit interaction and DNA-binding sites expected for an RNA polymerase beta prime subunit, but the *T. ingrica* sequence has several active-site substitutions, and neither has a complete catalytic site D–D–D sequence. The N-terminal domains resemble other beta prime sequences, but the C-terminal domains differ from each other and their genomic partners in the variable S13 region. The BOGUAY ORF has three TAACTGA units in forward orientation just upstream of its start codon, interleaved with two TTACTGA sequences, and three in reverse orientation within the ORF, one direct TCAGTTA repeat and a third unit separated by the related 7-mer TCAATTA (**Figure 6A**, below). These encode the amino acid sequence LSVINYQLS and fall within a variable region of the predicted beta prime N-terminal domain (Figure 4), which in the *E. coli* crystal structure is in a surface loop near the alpha II subunit (Murakami, 2013).

**Figure 5:**
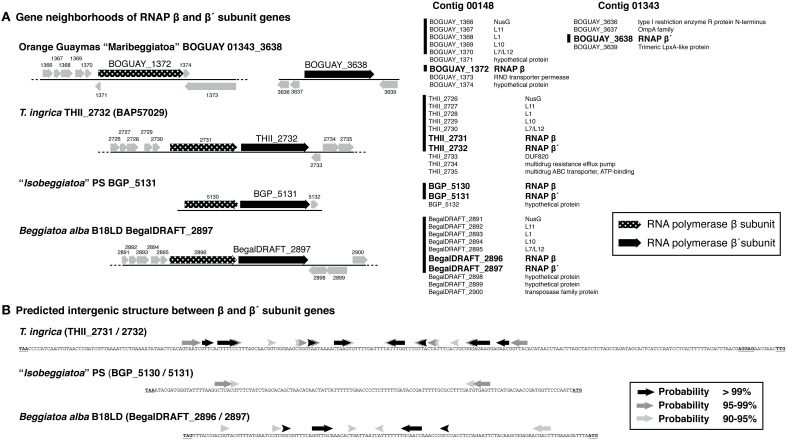
**Gene neighborhoods and intergenic sequence for putative RNA polymerase beta and beta prime subunit genes from ***Beggiatoaceae*** genome sequences. (A)** Gene neighborhoods are shown as cartoons (left) and lists (right). Vertical lines alongside lists indicate ORF sequences common to at least three species (the “Isobeggiatoa” PS genome is very partial). RNA polymerase subunit genes are in bold. **(B)** Predicted stem-loop structures in the beta/beta prime intergenic region are the first results from a minimum free energy calculation using the default settings of the MaxExpect algorithm from the RNAstructure Web Server (http://rna.urmc.rochester.edu/RNAstructureWeb/, Reuter and Mathews, 2010), with arrows shaded by the probability of the interaction.

The genomic context of the BOGUAY beta prime is unusual. Of the four *Beggiatoaceae* beta prime genes for which some surrounding sequence is available (Figure 5A), all but BOGUAY have beta and beta prime genes immediately adjacent, as do many if not most other bacteria (Dandekar et al., 1998). Upstream of the putative beta subunit gene, BOGUAY, *T. ingrica*, and *B. alba* each have a NusG and four ribosomal protein genes; the “Isobeggiatoa” PS contig does not extend upstream. Downstream, the BOGUAY beta and beta prime subunit genes have apparently become separated, being internal to separate contigs. Comparing the beta/beta prime intergenic regions in the other three species, that in *T. ingrica* is longer (338 nt) than those in *B. alba* and BGP (126 and 133 nt, respectively). It also includes a stronger potential stem-loop structure (Figure 5B), possibly a transcriptional terminator. One scenario is that the two genes became transcriptionally uncoupled in a common ancestor of *T. ingrica* and the BOGUAY strain, making the intergenic region a viable site for genomic rearrangements and introduction (by whatever mechanism) of TAACTGA repeats. If the putative beta prime variants are in fact expressed, perhaps the separation of beta and beta prime allows the levels of the three proteins to be separately regulated.

#### Predicted sensor proteins are immediately downstream of the putative secondary beta prime subunit genes

**Figure 6:**
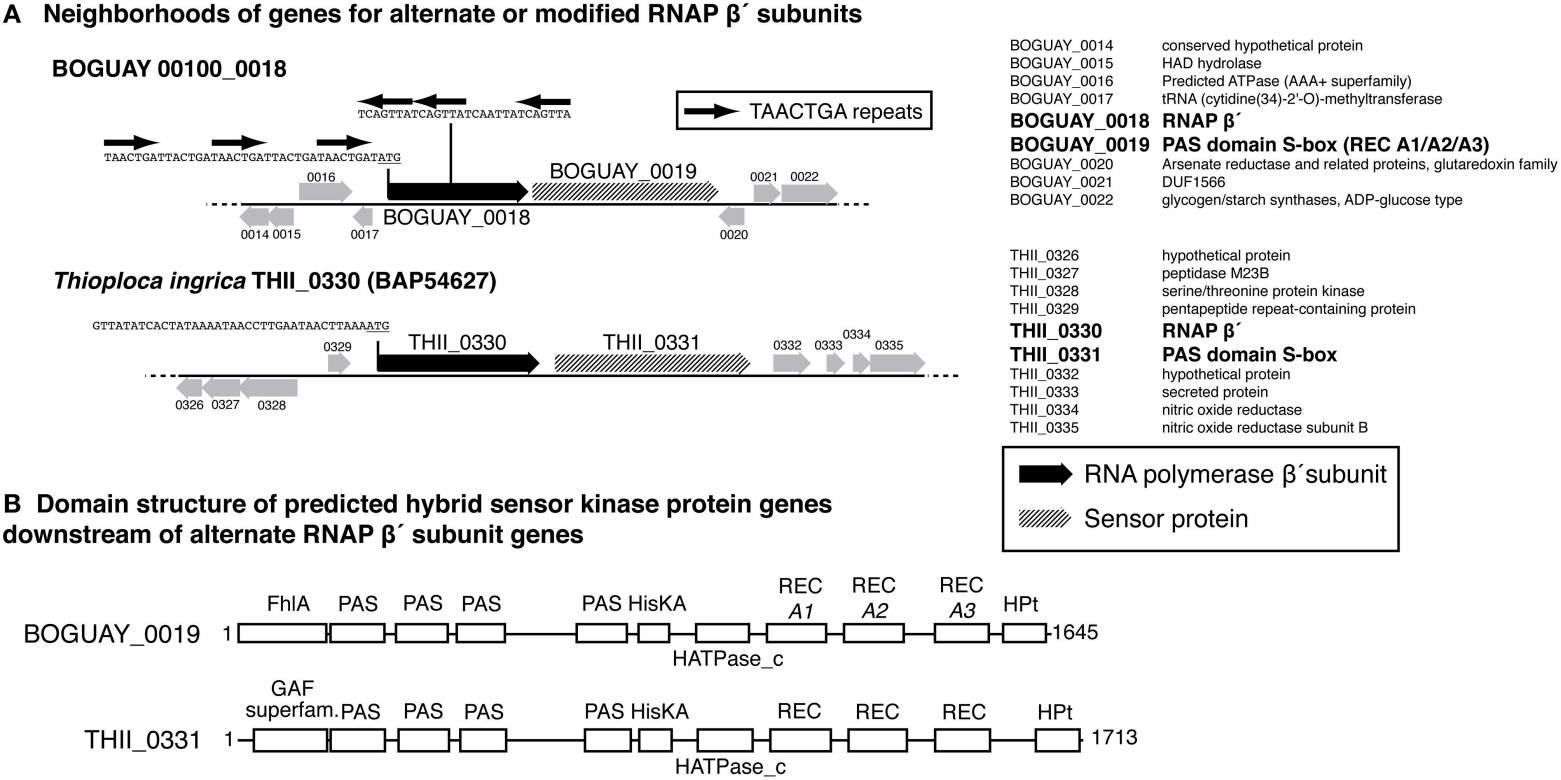
**(A)** Gene neighborhoods for putative alternate RNA polymerase beta prime subunit genes from the BOGUAY and ***Thioploca ingrica*** genome sequences. Gene neighborhoods are shown as cartoons (left) and lists (right). Positions of TAACTGA repeats within and upstream of BOGUAY 00100_0018 are indicated; the corresponding upstream sequence from *T. ingrica*, which has no repeats in this region, is included for comparison. **(B)** Predicted domain structures of putative downstream sensor proteins. Domains were identified in CDD (Marchler-Bauer et al., 2011).

Each of the variant beta prime genes is immediately followed by a predicted hybrid sensor kinase gene (Figure 6). These have nearly identical structures according to the Conserved Domain Database (CDD; Marchler-Bauer et al., 2011): a GAF-superfamily domain, four PAS domains, a histidine kinase, three REC domains, and an HPT domain. GAF domains, which include those in FhlA (formate hydrogen lyase transcriptional activator)-family proteins, bind and respond to cyclic-nucleotide second messengers (Aravind and Ponting, 1997). PAS domains are intracellular or periplasmic redox sensors responsive to various stimuli, including light and oxygen, with specificity determined partly by small-molecule cofactors such as a heme or flavin (Taylor and Zhulin, 1999; Kneuper et al., 2010). HisKA-HATPase_c (histidine kinase A—histidine-kinase-like ATPase) domains respond to sensor inputs by autophosphorylating on a histidine residue, which in turn typically phosphorylates a response regulator (REC) domain aspartate residue (Stock et al., 2000), changing its conformation and, for example, promoting dimerization and DNA binding. HPt (histidine-containing phosphotransfer) domains transfer phosphate groups to other proteins along phosphorylation cascades (Matsushika and Mizuno, 1998). Both the BOGUAY and *T. ingrica* putative sensor proteins are strongly predicted by PSORTb (Yu et al., 2010) to be inner-membrane proteins, by comparison with *E. coli* BarA, which was localized in a membrane proteomic survey (Daley et al., 2005). As is usual with the highly modular sensor proteins, neither has any other full-length matches in current databases, although each of the subdomains does. There is not yet enough known about sensor proteins to predict what stimuli these might respond to, or what their upstream and downstream interaction partners might be, but it can be hypothesized that they sense a condition in the periplasm and transmit that information to cytoplasmic elements via a phosphorylation cascade, which may directly or indirectly contact the variant beta prime.

## Summary and perspectives

### TAACTGA repeats may play different roles in different species

The draft genomes of Orange Guaymas “Maribeggiatoa” (BOGUAY) and *Cand*. “Thiomargarita nelsonii,” and to a lesser extent *T. ingrica*, contain an unusually high number of TAACTGA direct repeats, while close relative *B. alba* and apparently all but one other sequenced Gammaproteobacterium (*T. violascens*, also a sulfur oxidizer) have none at all. TAACTGA direct repeats were also found in Cyanobacteria, especially in species known for harboring long repetitive arrays, and in a few Bacteroidetes. This is consistent with earlier evidence for genetic exchange among these groups (MacGregor et al., 2013c), particularly the Cyanobacteria and some *Beggiatoaceae*, although no exchange mechanism is obvious as yet. Once introduced into a genome, whether by exchange or mutation, the tolerated sites and orientations for repeats will be determined by sequence characteristics such as length, coding potential, and propensity to form secondary structures, and by their interaction with existing cellular machinery. For the BOGUAY intergenic TAACTGA repeats, a plausible scenario is that they were recognized by an existing nucleic acid-binding protein—perhaps a ribosomal subunit, perhaps a protein that interacts with these—and over time a regulatory network evolved by selection for individuals with favorable protein interaction(s) and combinations of insertions. The original introduction may have happened in the common ancestor of a branch of the *Beggiatoaceae*, with different networks evolving (or not) in each subsequent lineage. The very long arrays in species such as *M. aeruginosa* and *G. forsetii* suggest a role in genome rearrangement may have evolved in these. Acquisition of additional genome sequences for the *Beggiatoaceae* may help illuminate this history.

Another possibility is that a TAACTGA-binding protein is the mobile element. On entering a new species, it could interact with pre-existing “good-enough” RNA or DNA sequences, with closer matches and useful locations evolving over time. Identification of repeat-binding protein(s) in the BOGUAY genome and evaluation of their inferred phylogeny and gene neighborhoods in other species could help in evaluating this model.

### TAACTGA repeats may play a role in translational regulation in the BOGUAY strain

In the BOGUAY genome, most of the TAACTGA repeats are in “forward” orientation immediately upstream of putative start codons and overlapping the expected ribosome-binding site, suggesting that they may have taken on a role in translational regulation in this species. Genes and ORFs lacking recognizable Shine-Dalgarno sequences are prevalent in BOGUAY and many other bacterial genomes (Ma et al., 2002), including such highly expressed genes as the *E. coli* ribosomal protein S1 gene (*rpsA*; Aseev and Boni, 2011); in BOGUAY, only a small proportion of these are preceded by TAACTGA repeats. Possibilities for the translational role of the BOGUAY repeats, not all mutually exclusive, include:

- Canonical BOGUAY ribosomes are able to bind efficiently enough to the repeats for production of even highly translated proteins, despite the absence of sequence complementary to the 16S rRNA.
- Ribosomes with different subunit compositions—in particular, those lacking S1—may have different binding sites, as already recognized for leaderless mRNAs; this could include TAACTGA repeats.
- Repeats may be recognized by some other RNA-binding protein (e.g., a Csp-like one), which then recruits ribosomes.
- Repeats are irrelevant, these genes are translated like leaderless mRNAs by ribosomes lacking S1.

### Possible function of second RNA polymerase beta prime subunit-like proteins in BOGUAY and *Thioploca ingrica*

Another unusual feature of the BOGUAY genome is a second RNA polymerase beta prime-like ORF, also found in *T. ingrica*, and immediately upstream of multisensor kinases in both. In BOGUAY, this putative alternate or modified gene is both preceded by and contains TAACTGA repeats. The BOGUAY genome has the additional peculiarity that the beta and “normal” beta prime genes are not adjacent, but rather internal to separate contigs. Assuming the beta prime-like gene is expressed, one possibility is that it associates with other RNA polymerase subunits, forming either a functional or a non-functional complex: the absence of key catalytic residues suggests it would be non-functional, but this would need experimental testing. This is somewhat supported by the physical separation of the beta and beta prime genes in BOGUAY, and their possible transcriptional separation in *T. ingrica*: if two proteins are competing for the beta prime role, it may be beneficial to regulate their production separately from that of their common partners. In BOGUAY, the TAACTGA repeats upstream of the beta prime-like ORF suggest that it may be part of their putative global regulatory network.

### Perspectives

Experimental tests of these ideas will be challenging in an uncultivated, difficult to collect species. Some basic questions may be answerable by transcriptomic analysis of samples collected from different Guaymas Basin sites and/or preincubated under different conditions (temperature, oxygen, sulfide). Are ORFs preceded by repeats up- and downregulated in concert? Is the second beta-prime like ORF transcribed, and if so, under what conditions? How does its expression pattern compare with that of other RNA polymerase subunit genes? There are also indications from the partial “Isobeggiatoa” genome sequences that the more accessible Baltic Sea *Beggiatoaceae* may have similar repeat distributions. *In vitro* identification of repeat-binding proteins might be possible from total mat protein preparations, or by heterologous expression and isolation of cloned (or synthesized) genes for candidate proteins.

## Funding

The Guaymas Basin project was funded by NSF OCE 0647633. Genome sequencing was performed by the J. Craig Venter Institute, with funding from The Gordon and Betty Moore Foundation Marine Microbial Genome Sequencing Project. The use of RAST was supported in part by the National Institute of Allergy and Infectious Diseases, National Institutes of Health, Department of Health and Human Services (NIAD) under contract HHSN266200400042C.

### Conflict of interest statement

The author declares that the research was conducted in the absence of any commercial or financial relationships that could be construed as a potential conflict of interest.
