# Supplementary material for: Abundant Intergenic TAACTGA Direct Repeats and Putative Alternate RNA Polymerase β′ Subunits in Marine Beggiatoaceae Genomes: Possible Regulatory Roles and Origins
Source: Front Microbiol. 2015 Dec 16;6:1397. doi: 10.3389/fmicb.2015.01397 (PMC4679880; doi:10.3389/fmicb.2015.01397)
Supplement: Supplementary file 1 [file Presentation1.PDF]

Supplemental files for:

Abundant intergenic TAACTGA direct repeats and putative alternate RNA polymerase β´ subunits in marine *Beggiatoaceae* genomes: possible regulatory roles and origins

Barbara J. MacGregor
Department of Marine Sciences
3202 Venable Hall, CB#3300
University of North Carolina – Chapel Hill
Chapel Hill, NC, 27599, USA

bmacgreg@unc.edu

Supp. Fig. 1. BOGUAY ORFs with internal TAACTGA repeats in reverse orientation 3

Supp. Fig. 2. TAACTGA repeats in the neighborhood of putative *Beggiatoaceae* ribosomal protein genes 4

Supp. Fig. 3. TAACTGA repeats in the Bacteroidetes *Aequorivita sublithincola* DSM 14238 and *Gramella forsetii* KT0803 5

Supp. Table 1. BOGUAY repeat-containing regions by position, orientation, and COG category of downstream ORF 6

Supp. Table 2. TAACTGA repeat distribution in *Beggiatoaceae* and other genomes 16

Supp. Table 3. Sample of TAACTGA repeats within the *Microcystis aeruginosa* NIES-843 genome 17

Supp. Table 4. TAACTGA repeats in *Cyanothece* strains PCC 8801, 8802, and 7424 21

Supp. Table 5. TAACTGA repeats in *Flexibacter litoralis* DSM 6794 and *Paludibacter propionicigenes* WB4 24

Supp. Table 6. TAACTGA repeats in *Elizabethkingia anophelis* NUHP1 25

Supp. Table 7. XisH and XisI annotations and BOGUAY_0693 orthologs in species with TAACTGA repeats 26

Supp. Table 8. Partial census of predicted Shine-Dalgarno sequences in the BOGUAY genome 27

Supp. Table 9. First 100 blastp results for the sequence SVISYQLSV 28

Supp. Table 10. BOGUAY ORFs containing CSP-CDS domains 32

References 33

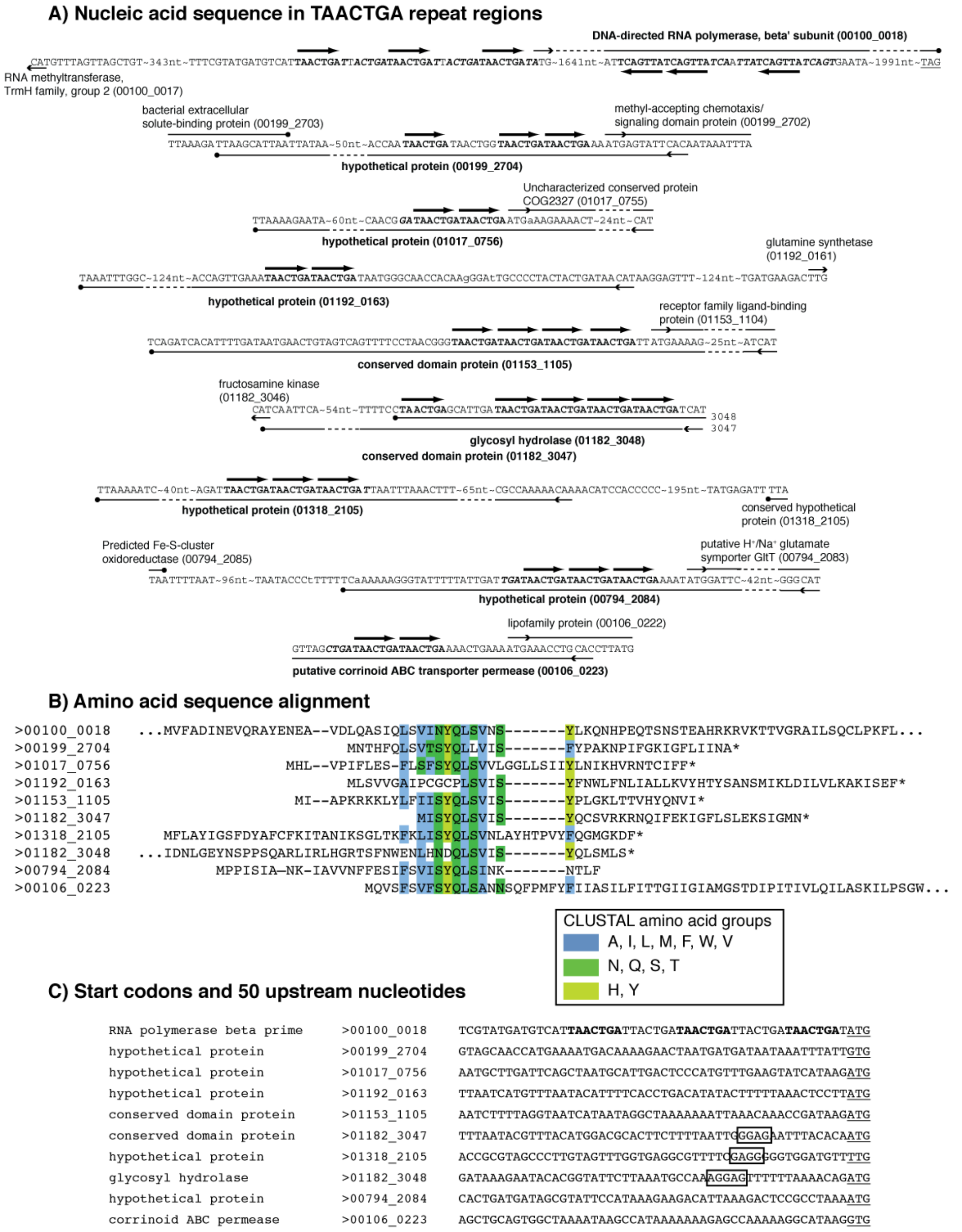

Supplemental Figure 1. BOGUAY ORFs with internal TAACTGA repeats in reverse orientation.

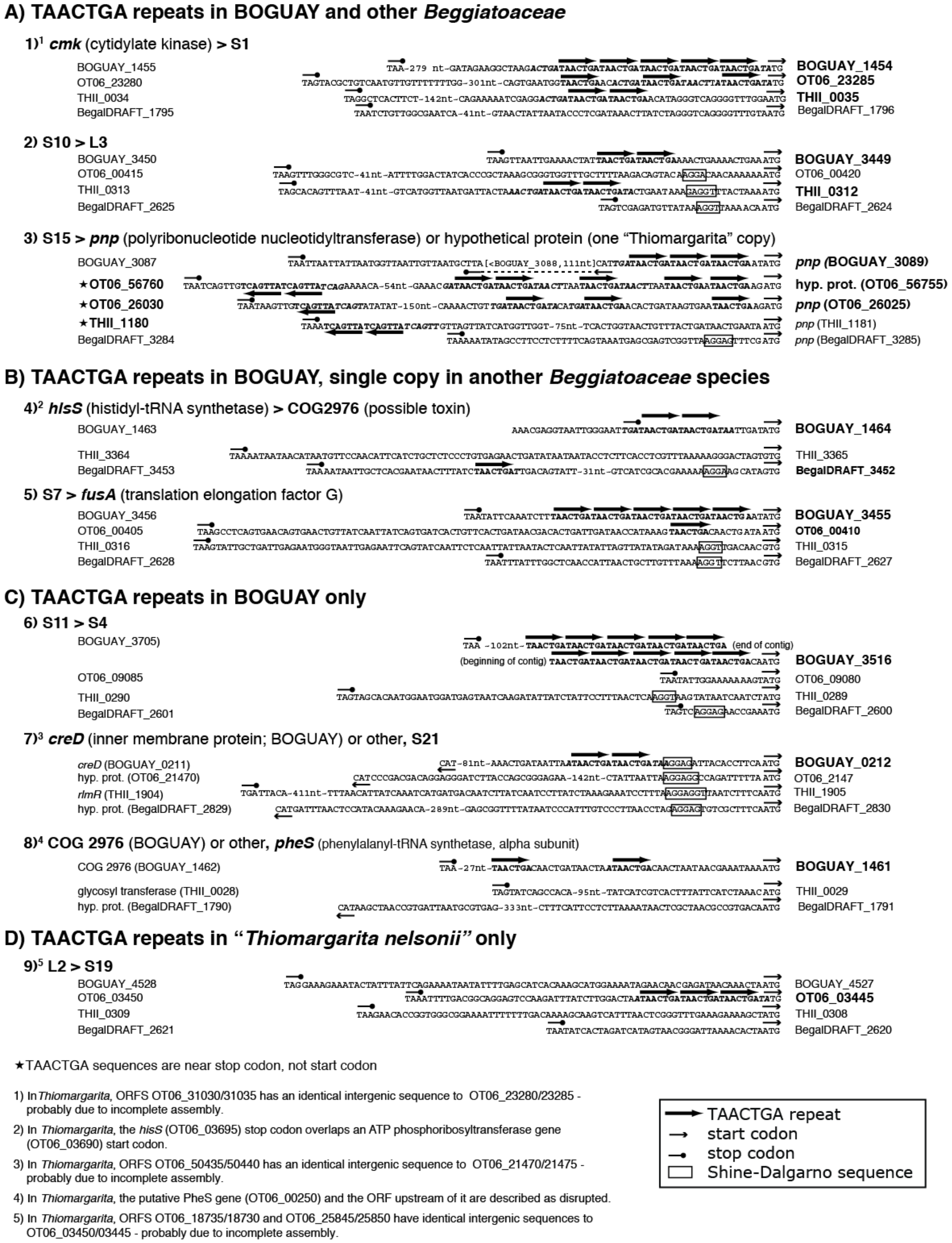

Supplemental Figure 2. TAACTGA repeats in the neighborhood of putative *Beggiatoaceae* ribosomal protein genes. Corresponding regions for species without repeats are included for comparison.

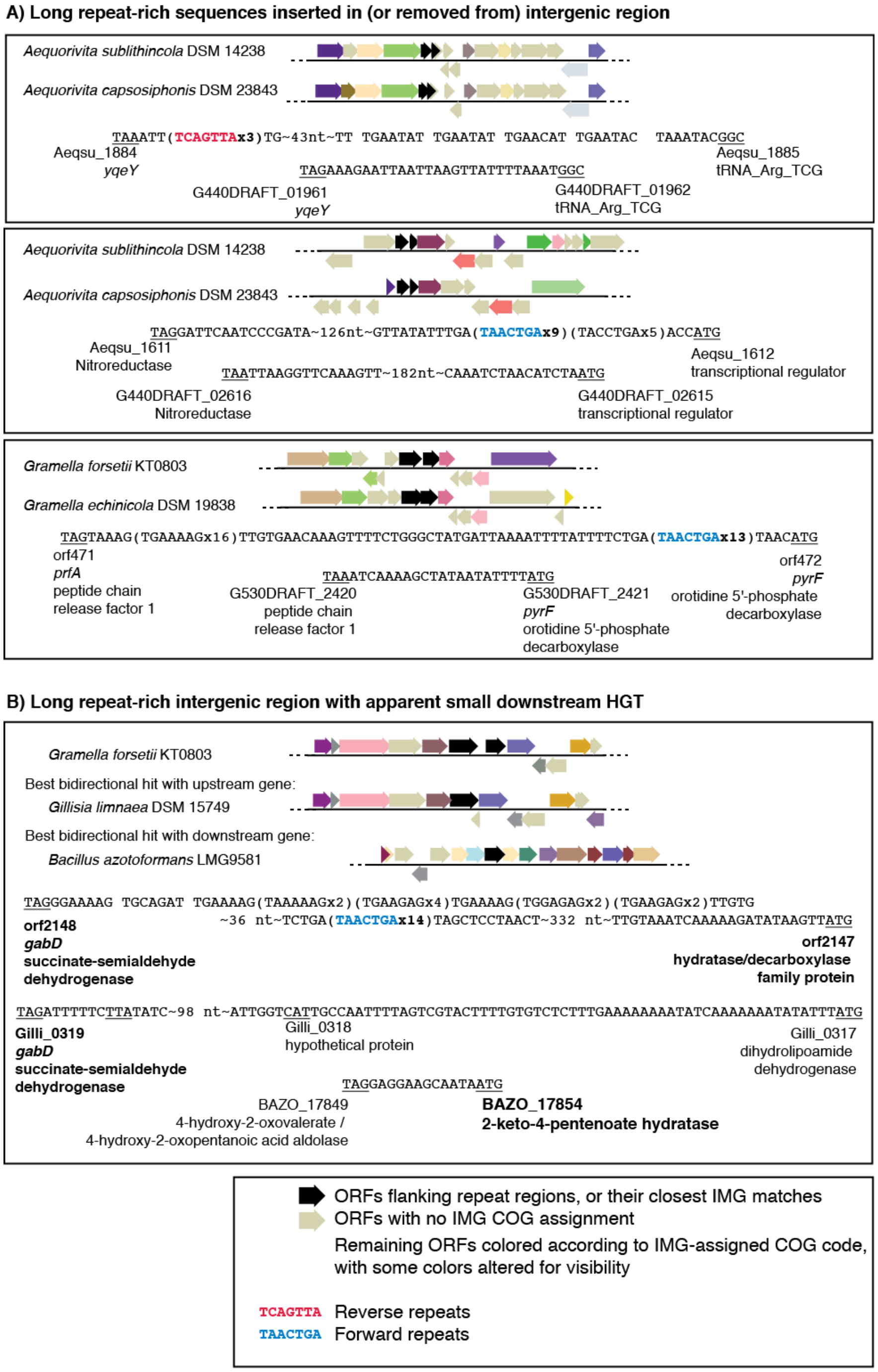

Supplemental Figure 3. TAACTGA repeats in the Bacteroidetes *Aequorivita sublithincola* DSM 14238 and *Gramella forsetii* KT0803. Within each box, the repeat-containing sequence is illustrated at the top, and the closest database match(es) for the flanking ORFs below that, followed by illustrations of the intergenic repeat-containing sequences in the same order.

Supplemental Table 1. BOGUAY repeat-containing regions by position, orientation, and COG category of downstream ORF. (A) TAACTGA repeats upstream of ORFs with no recognizable Shine-Dalgarno sequences in the BOGUAY genome. Sequences are organized by the COG category of the downstream ORF (if any) and then by ORF number. **(B) Repeats at the end of contigs with no downstream ORFs. (C) TAACTGA repeats in other orientations.** COG functional categories are as follows: A: RNA processing and modification; B: Chromatin structure and dynamics; C: Energy production and conversion; D: Cell cycle control and mitosis; E: Amino acid metabolism and transport; F: Nucleotide metabolism and transport; G: Carbohydrate metabolism and transport; H: Coenzyme metabolism; I: Lipid metabolism; J: Translation; K: Transcription; L: Replication and repair; M: Cell wall/membrane/envelop biogenesis; N: Cell motility; O: Post-translational modification, protein turnover, chaperone functions; P: Inorganic ion transport and metabolism; Q: Secondary structure; T: Signal transduction; U: Intracellular trafficking and secretion; Y: Nuclear structure; Z: Cytoskeleton; R: General functional prediction only; S: Function unknown

(A)

Upstream ORF	Repeat region sequence	Downstream ORF		Downstream COG category
hypothetical protein	TAGCAGGATA~62nt~AACACTACGCCTGATAACTGATAACTGATAACTTATAACTAATTATG	00285_1236	putative NADH-quinone oxidoreductase chain 5	C
Predicted Fe-S-cluster oxidoreductase 00794_2085	TAATTTTAAT~96nt~TAATACCCtTTTTTCaAAAAAGGGTATTTTTATTGATTGATAACTGATAACTGATAACTGAAAATATGGATTC~42nt~GGGCAT 00794_2084	00794_2083	putative proton~sodium-glutamate symport protein GltT	C
Uncharacterized protein conserved in bacteria COG3198	CATTTTAAGTTATCAGTTATATGTTATCAGTTA~214nt~CATATATTCAACATAACTGATAACTGATAACTGAAATG	01148_1376	3-isopropylmalate dehydrogenase	C
Uncharacterized protein conserved in bacteria COG 4222	(>100 bp to next ORF) GAAAAAAATTGAACCTCGAAAAAGTAATTTTTACTGATAACTGATAACTGATAACTGATAACTGAATATG	01191_1496	NADH dehydrogenase subunit M	C
hypothetical protein	CATAAACTG~72nt~TATAACGGATAACTGATAACTGATATATG	00326_4922	septum formation protein Maf	D
Predicted permeases COG0795	(>100 bp to next ORF) AAGGTTTTGAATTTTATCAAAAAATTGATATTTGATGCCATACTAACTAATAACTGATAACTGAATATG	00822_0334	cytosol aminopeptidase family, catalytic domain protein	E
hypothetical protein	(>100 bp to next ORF) CAAAGGTTAATATTAACCGAACCTTGCTTGTGTTAAAAACTGATAACTGATAACTGATAACATTCATG	00701_1059	homoserine O-acetyltransferase	E
NnrS protein	(>100 bp to next ORF) TCAACTTTTAATAAATATTGATAACTGAACACTGATAACTGAAATG	00559_2565	argininosuccinate lyase	E
glycine cleavage system T protein	TTTTTAACTGATAACTGATAACTGATAATG	00883_3308	glycine cleavage system H protein	E
dihydrodipicolinate synthetase family protein	CATACATTTTAGC~93nt~TGTTTTGAAAATAACTGATAACTGATAACTGAGATAACTGATG	01092_1325	aminotransferase	E
S-adenosylmethionine:tRNA ribosyltransferase-isomerase *queA*	TAAGATGAAGTAACTGATAACTGATAACTGATAACTGACATG	00153_2342	asparate kinase, monofunctional class	E
putative lipoprotein	GCCCTATACCGACGGAAAAACAGCCACTGAATAATTGATAACTGATAACTGATAACTGATAACTGAAAAAATG	00862_3488	diaminopimelate decarboxylase (lysine biosynthesis)	E
putative thiamine-phosphate pyrophosphorylase	TAAGCTGATAACTGATAACTGATAACTGATAACTGATAACTGAAATG	01182_3039	cysteine desulfurase DndA	E
conserved domain protein	TCAGATCACATTTTGATAATGAACTGTAGTCAGTTTTCCTAACGGGTAACTGATAACTGATAACTGATAACTGATTATGAAAAG~25nt~ATCAT	01153_1104	receptor family ligand-binding protein	E
cation diffusion facilitator family transporter (further upstream)	TAAATTTGGC~124nt~ACCAGTTGAAATAACTGATAACTGATAATGGG~29nt~TAACATAAGGAGTTT~124nt~TGATGAAGACTTG	01192_0161	glutamine synthetase, type I	E
leucine Rich repeat protein	CATATTATTT~99nt~TATTAACCGATAACTGATAACTGATTAACTGAAATG	00016_1748	carA carbamoyl-phosphate synthase, small subunit	E, F
phosphoglycolate phosphatase, bacterial	(>100 bp to next ORF) AAAAAATACCTTGCACCTACATTTTAACTGATAACTGATAACTAATAACTGACAACTAATACATG	00136_0642	chorismate binding enzyme	E, H

TAACTGA repeat
start codon
stop codon
tRNA begins

Upstream ORF	Repeat region sequence		Downstream ORF	Downstream COG category
PAS domain S-box	TAAGGAACGCACAACTAGGGCTTGCCCTTAATTAATACTGATAACTGATAACTGATG	00470_1294	dUTP diphosphatase	F
PAS domain S-box	TAATTGTTAACTGATAACTGACATG	00806_3000	thymidylate synthase	F
calcium-translocating P-type ATPase, PMCA-type	CATATAAGCC/96nt/GGTTCTGAAAACTGATAACTGATAACTGATATAATTATG	00423_4037	dihydroorotate dehydrogenase (UMP biosynthesis)	F
organic solvent tolerance protein	CATTTTAAATTATTTGTGAATGATGTAGTCGTTAAATTTTTTTATTTATCTGAAAACTATAACTGATAACTGACAACTGATAACTGATG	01051_2210	phosphoribosylaminoimidazole-succinocarboxamide synthase	F
acetyl-CoA carboxylase, carboxyl transferase, beta subunit	(>100 bp to next ORF) CAAAACTGATAACTGATAAAGCAACCACAAAGAATTGCCCCCCACTAACTGACAACTGATAACTGATAACTGATAATG	01050_3532	ribonucleoside-diphosphate reductase, alpha subunit	F
formate/nitrite transporter	TAACCTGAATTAAGGGCGAACACGAGGGCGAACACGCAGGTTCGCCCCTACTTTAACTGATAACTGATAACTGATAATTAAATATG	00256_1384	nucleoside diphosphate kinase	F
PTS system fructose IIA component	TAAATTTTCACGCAAAAGTTTTGGCGTGAATACTGATAACTGAATAACTGATAACTGACATG	00136_0634	phosphocarrier, HPr family	G
5-formyltetrahydrofolate cyclo-ligase	CTGATAAACTAATTGTGAACTGATAACTGATAACTGATAACTGATTATG	01160_3498	inositol monophosphatase family protein	G
electron transport complex, RnfABCDGE type, B subunit	TGAGAAAAATATCAAGCCCCCAGTTATGCTCGGTTTCTATAATTTGCTTTGACATTATGGGAACTGGGAACTGATAACTGATAACTGATAACTGACAACTGATAATG	00072_1962	1,4-alpha-glucan branching enzyme GlgB	G
UDP-N-acetylmuramate: L-alanyl-gamma-D-glutamyl-meso-diaminopimelate ligase	TAATTTGGAAATTGGGAATTGATAACTGACAACTGATAACTGAATATG	00822_0353	aromatic acid decarboxylase	H
fructosamine kinase	TAAGAAAATTTAATGATAATTGAATAAAATGACTGATAACAGATTTATTAAATATACTATAACTGATAACTGATAACTGAATG	01182_3045	adenosylmethionine-8-amino-7-oxononanoate transaminase	H
CDP-diacylglycerol--glycerol-3-phosphate 3-phosphatidyltransferase	(>100 bp to next ORF) ATAATTTTCTAAAAATCTTAACTGATAACTGATAACTGAATTATG	00478_0861	molybdenum cofactor biosynthesis protein B	H
ABC transporter, ATP-binding protein	AATTGATAACTGATAACTGATAACTAATAACTGATAAAATG	00127_3139	methyltransferase domain protein	H
uroporphyrinogen-III synthase	TCACTGTTTTGAAA/126nt/GGTCACTTTTGATAACTGATAACTAATAACTGATAACTGATACATG	00362_1743	glutamyl-tRNA reductase	H
riboflavin biosynthesis protein RibD	TGAACAGTTATCAGTTACCAATTAC/81nt/ACTTGTAACTGATAATTAATAACTGATAACTGAAAAACATG	00397_1678	riboflavin synthase, alpha subunit	H
hypothetical protein	TAATTTTTTATCTGTGTTCAGTTATCAGTTGATAATAATTAATACCCTACATAACTGATAACTGATAACTAATAACTGATAACTGAAAAATATG	00163_1016	3,4-dihydroxy-2-butanone-4-phosphate synthase	H
type I secretion target GGXGXDXXX repeat (2 copies)	TAAAGCAGCCCTTAAAAATCTGATTTTTTATAACGTTCTAACTGATAACTGATAACTGATAACTGATG	00570_2192	beta-hydroxyacyl-(acyl-carrier-protein) dehydratase FabA	I
L-aspartate oxidase	TGATTAATTGTGAAATGCAGTAACGAAGTCGTTGATAACTGATAACTGATAACTGATAACTGATTATG	00239_2139	oxidoreductase, short chain dehydrogenase/reductase family protein	I, Q, R

91

Upstream ORF	Repeat region sequence	Downstream ORF		Downstream COG category
RDD family protein	TAGAAATCAAGTTTTTCAAAAGGTTTTAGTAAAATAACTGATAACTGATAAAAAATTG	00100_0010	Sua5/YciO/YrdC/YwlC family protein (translation factor?)	J
ribosomal protein S10p/S20e	TAAGTTAATTGAAAACTATTAACTGATAACTGAAAACTGAAAACTGAAATG	00458_3449	50S ribosomal protein L3 RplC	J
major cold shock protein CspA	(>100 bp to next ORF) ATACTATTTTTGCCAAAAATAGTATTTCTAATAATCGTTTACGGGTAACTGACAACTGACAACTGATAACTGAATCTATG	01308_0438	glycyl-tRNA synthetase, alpha subunit	J
hypothetical protein	TAAGACACTATCATAGTAATTGTGAATTATTAACTGACAACTGATAACTAATAACTGACAACTAATAACGAAATAAAATG	00726_1461	phenylalanyl-tRNA synthetase, alpha subunit	J
ribosomal protein S15	TAATTAATTATTAATGGTTAATTGTTAATGCTTAATGTTT~97nt~CCATTAACCATTGATAACTGATAACTGATAACTGAATATG	00969_3089	polyribonucleotide nucleotidyltransferase	J
Micrococcal nuclease (thermonuclease) homologs	CATATGTGTTTTTAGACTTTCAGTGGTATAATAAATGCGTTCGCCAATAATTACATGCTCCAAAGGCGAAATAAAAATTAACTGATAACTGATAACTGAATCATTATG	01090_3690	lysyl-tRNA synthetase	J
tRNA (guanine-N(7)-)-methyltransferase	(>100 bp to next ORF) TTATTTTCACGCGTTTTTGCGTGAAACTGATAACTGATAACTGATAACTGATAAGTGAATAACAATG	00780_1605	Sua5/YciO/YrdC/YwlC family protein (Putative translation factor (SUA5))	J
prenyltransferase, UbiA family	(>100 bp to next ORF)GTTTTTTGAAAAATTTAACTGATAACTGATAATTAATAACTAATAACTGATAACTGACAAAATG	00153_2331	translation initiation factor IF-1	J
cytidylate kinase	(>100 bp to next ORF) ATGATAGAAGGCTAAGACTGATAACTGATAACTGATAACTGATAACTGATAACTGATATG	00726_1454	ribosomal protein S1	J
ribosomal protein S7 RpsG	TAATATTCAAATCTTTAACTGATAACTGATAACTGATAACTGATAACTGAATATG	00458_3455	translation elongation factor G	J
(beginning of contig)	TAACTGATAACTGATAACTGATAACTGATAACTGACAATG	00680_3516	ribosomal protein S4	J
30S ribosomal protein S11	TAATCAGTGAGCCGTTATCAGTTATCAGTGGTTTAGAA~54nt`TTCTTACTTGAGATAACTGATAACTGATAACTGATAACTGATAACTGA	none	(end of contig)	
RNA methyltransferase, TrmH family, group 2	(>100 bp to next ORF)TTTTCGTATGATGTCATTAACTGATTACTGATAACTGATTACTGATAACTGATATG	00100_0018	DNA-directed RNA polymerase, beta' subunit	K
PDZ domain (Also known as DHR or GLGF)	(>100 bp to next ORF) TCCTGTACAAAGCGTTTAAAATATAACTGATAACTGAAATAATTGATAACTCA	00301_1145	Stage II sporulation protein E	K, T
Diacylglycerol kinase catalytic domain	TAGGTTCATTGTGAACTGATTTTCACGCAAACGTTTTTGCAGCCGCAAAAACGTTTGCGTGAAACTGAGATAACTGATAACTGATAACTGAAAAAGTG	00325_4420	DNA repair protein RecN	L
CTP synthase	(>100 bp to next ORF) TGCTAATGGTTAAGTTTTAGCCATAAATTTTAACTGATAACTGATAACTGATAACTGACATG	00349_1720	nudix-type nucleoside diphosphatase, YffH/AdpP family	L, R

Upstream ORF	Repeat region sequence	Downstream ORF		Downstream COG category
signal recognition particle protein	TAATTGTGAATTAATAACTGATAACTGATAATTTATAATG	00100_0060	glycosyltransferase, group 1 family protein	M
hypothetical protein	(>100 bp to next ORF) CTGATAATTGATAACTGA TAAT TGATAACTGA TAATTGACTTAAAAATG	00136_0638	N-acetylmuramoyl-L-alanine amidase	M
hypothetical protein	(>100 bp to next ORF) TTATTATATATAAACCTGACAGGATTCATTAACCATTAACCCTTAAT ATAACTGATAACTGAAAATGCTTT TAACTGA	00009_2815	UDP-N-acetylmuramoyl-L-alanyl-D-glutamate--2, 6-diaminopimelate ligase	M
tetratricopeptide repeat protein	TAATTAACGAACACACAGTTACCACAGGTTCCACCTGTGGTAACTTGAAGAATGCTGATAACTGATAACTGATAACTGATG	00824_3097	outer membrane lipoprotein LolB	M
hypothetical protein	TAAGCAGATTAAGGGCAGACACGCTTGTAGGGGCGAACCTGCGTGTTCGCCCTGATAACTGATAACTGATAACTGATAATG	00017_1809	efflux ABC transporter, permease protein	M
conserved domain protein	(>100 bp to next ORF) TTTAAAGAAAAATAAGAATGACTCAAATTCAAGTG GATAACTGATAACTGATAACTGATAACTAATAATTGAAATG	00360_3387	methyltransferase domain protein	M
putative oxygen-independent coproporphyrinogen III oxidase	CATAAATTAAACATGATTGATAACTGATAACTGAAATAACTGAACTATG	01104_3430	UDP-glucose 4-epimerase	M
bacterial extracellular solute-binding protein (00199_2703)	TTAAAGATTAAGCATTAATTATAA~50nt~ACCAATAACTGATAACTGGTAACTGATAACTGAAAATGAGTATTCACAATAAATTTA<br>00199_2704	00199_2702	methyl-accepting chemotaxis protein signaling domain protein	N, T
iron-sulfur cluster assembly protein IscA	TAAAGAACTGATAACTGGTAACTGATAACTGAAATG	00136_0667	Fe-S protein assembly co-chaperone HscB	O
Fe-S protein assembly co-chaperone HscB	TGAAACGTAGTAATTATGACTGAATAACTGA TGACTGAATAACTGATG	00136_0668	Fe-S protein assembly chaperone HscA	O
conserved hypothetical protein	(>100 bp to next ORF) GCAAGAACTTAACTAAATAACTGA TAAT TGATAACTGA AAAATG	00162_0485	putative glycoprotease GCP	O
tRNA-Ser-CGA	CCATATATGGA~68nt~AAATAACACTGATAACTGA TAACTTA TAACTGATAACTGA TATG	00031_3797	leucyl/phenylalanyl-tRNA--protein transferase	O
Cytochrome c-type biogenesis protein CcmE	TAATGAAAACATAATTGATAAAGGGCGAACACTTATGTTCGCCCCTACATACGTAATGATAACTGATAACTGATAACTGAAATATG	00016_1753	Cytochrome c-type biogenesis protein CcmF	O
putative lipoprotein	(>100 bp to next ORF) AAAAAAAAAGTCCAAAAATTACCCGTTAGTTATGTAGTGAATT TGATAACTGATAACTGATAACTGATAACTGATAACTGATAAAATG	00199_2705	ATPase family associated with various cellular activities (AAA)	O
cytochrome C1 family protein	TGATTCGGTTATCAGTTATCAGTTAATTGCGGC~65nt~TTAGAATAACTGATAACTGATAACTGATAATTGGTAACGGATAACTGATCACTGATAACTACGGATAACTACGGATAACTGATG	01232_0393	putative stringent starvation protein A	O
kinase domain protein	CATAATTTTAA~96nt~AATTATAACAATT TAACTGATAACTGA TAATG	00676_3756	ABC-type transport organic solvent resistance, periplasmic component	Q

Upstream ORF	Repeat region sequence	Downstream ORF		Downstream COG category
cytosol aminopeptidase family, catalytic domain protein	(>100 bp to next ORF) CTATAGTATTTAAATAAATTTTCATGACTTAGATACAATAAAGAACTTTACCATAACCATAAATAACTGATAACTGATTATT	00822_0335	Predicted permeases COG0795	R
putative corrinoid ABC transporter permease	GTTAGCTGATAACTGATAACTGAAAACTGAAAATGAAACCTGCACCTTATG	00106_0222	lipofamily protein	R
**COG3222, DUF2064**	AGATGACAACTGATAACTGATAACTGATAACTGATATG	00162_0503	homoserine kinase, ThrB	R
hypothetical protein	(>100 bp to next ORF) GCTTTATACTGAAATAATTGTGAAACGAAATAACTGATAACTGATAACTGATAACTGGTAGCTGAAATGATG	00692_3330	putative lipoprotein	R
Uncharacterized conserved protein DUF3620	(>100 bp to next ORF) GGGCAAACTGATAACTGATAACAGATAACTGATAACTGATAACTGATAACAAAATAATG	00628_3544	FxsA cytoplasmic membrane protein	R
phosphoglucomutase/phosphomanno-mutase, alpha/beta/alpha domain III	(more than 100 bp to next ORF) CCACCCTATATTTTTACTGATAACTAATAACTGATAACTGAAGAGCCGTTATG	00138_2545	putative toxin-antitoxin system, antitoxin component	S
D,D-heptose 1,7-bisphosphate phosphatase	CGCAGCGACGAATTTAATTTTAAACTGATAACTGATAACTGACATG	00203_2410	Uncharacterized protein conserved in cyanobacteria	S
histidyl-tRNA synthetase	TTATCTCAAATCGTTAGAATTTGGAAACGAGGTAATTGGGAATTGATAACTGATAACTGATAATTGATATG	00726_1464	Uncharacterized protein conserved in bacteria	S
electron transport complex, RnfABCDGE type, E subunit	GCCCTAAAAAATGCCATAGATAAACGTTTATAACTGATAACTGATAATTGAACAATG	01035_2232	Uncharacterized protein conserved in bacteria COG3122	S
hypothetical protein	TTAAAAGAATA~60nt~CAACGGATAACTGATAACTGAATGaAAGAAAACT~24nt~CAT	01017_0755	Uncharacterized conserved protein COG2327	S
penicillin-binding protein, 1A family	TAATTGTGAAACGCAGTGACGAAGTCATAGATAACTGATAACTGATAACTGATAACATG	00632_0130	acyl-phosphate glycerol 3-phosphate acyltransferase	S
putative lipoprotein (PilJ)?	CATTGATTA~59nt~GAAAAGTCTACTGATAACTGATAACTGATAACTGATAACTAATATG	00675_4726	conserved hypothetical protein TIGR00255	S
ATP-dependent protease HslVU, ATPase subunit	TAACTTTTCAGTTACCAGTTATCAGTTATTCAGTTATCAATTAGCCTTTCG~91nt~TTTATTATGATAACTGAACACTGATAACTGATAACTGAACACTGATAACTAACTGAAAAACATG	00335_1769	Uncharacterized protein conserved in bacteria DUF971	S
Phosphate-selective porin	(>100 bp to next ORF) CATAAAGCGATAATTAAACTGATAACTGATAACTGAAATG	01092_1332	nitrogen fixation negative regulator NifL	T
ABC transporter, ATP-binding protein	TGAAATGGGTTCATTGCGTCATTTATAACTGATAACTGATAACTAAATAATG	00847_4025	protein phosphatase 2C	T
recombination associated protein RdgC	TAGGAATGC~75nt~GCCTACTAATTTATAACTGATAACTAATAACTGAATTG	00570_2188	PhoH family protein	T
hypothetical protein	CATGCCGAAG~76nt~TTGTTTGATAACTGATTTTTACTGATAACTGATAATTGATAATTAATG	00239_2130	response regulator receiver domain protein	T
adenylate and guanylate cyclase catalytic domain protein	aAAAAACTGATAACTGATAACTGATAACTGATAACTGATAAACATG	00391_1539	hemerythrin HHE cation binding domain protein	T
ATPase, histidine kinase-, DNA gyrase B-, and HSP90-like domain protein	AAGGATGAACTGATAACTGATAACTGATAACTGATAACTGAAAAAaATG	00163_1011	response regulator receiver domain protein	T

Upstream ORF	Repeat region sequence	Downstream ORF		Downstream COG category
Tol-Pal system-associated acyl-CoA thioesterase	TAACTGATAACTGATAACTGATAACTGAAAAATG	00647_3823	protein TolQ	U
DsbA-like thioredoxin domain protein	(>100 bp to next ORF) ACCAATTTCTGTCGCCCAACAAATTTTAACAACTTATCTCATTTACATACATTACTGATAACTGATAACTGAGATG	00133_3591	ABC transporter, ATP-binding protein	V
bile acid transporter family protein	(>100 bp to next ORF) AAAGGATTTTTCAACCCTTAATTCGCATTATTATAACATAGGAAAAACCAGTCGTTAAAATAACTGATAACTGATTATG	01148_1353	putative lipoprotein	(none assigned)
conserved domain protein (seems to be direct repeat of 00762_4276)	TAAACGGCTG~100 nt~AGGGCAACCAATCGGGCTTGCCCTACTAACTGATAACTGATTATG	00762_4276	Protein of unknown function (DUF1703)./Predicted AAA-ATPase	
ABC transporter, ATP-binding protein	(>100 bp to next ORF) CGCCAGTGCATAAATACTACCCAATATCATTGGTAACTGATAACTGATA TATG	00162_0514	conserved domain protein	
WD domain, G-beta repeat protein	TAATCTCGAATTTATAACTGATAACTGATAACAGAATG	00394_1819	hypothetical protein	
conserved domain protein	(>100 bp to next ORF) AGGTATTGGTATAGAAATCCAATGTAATAAAAATCGGATTTATTTTGAACTTAACTGATAACTGATAACTTATATG	00463_3785	conserved hypothetical protein DUF4124	
putative membrane protein	CATGGTTATTTTTGATAATTGTGAATTGTGAATTTATAATTATAACCAGTCTGATAACTGATAACTGATAAATGAAATGATG	00024_0711	putative membrane protein	
hypothetical protein	TGATTTTGTTTTTATGGAA~93nt~GGAACAATACAACTGATAACTGATAACTGATTGGAAAAATTTATG	00871_2433	peptidase, M16 (pitrilysin) family protein	
RDD family protein	CATAGTTG~80nt~CTTTTTAGTAATAACTGATAACTGATAATTGAAACAATG	00100_0008	ATP-dependent Clp protease adaptor protein ClpS	
tRNA-Thr-CGT	(>100 bp to next ORF) CATTTATCAAAGTGACTAATTGTTAGCTAATAACTGATAACTGATAATTGATAAAAGCTATG	00901_3212	PAS domain S-box	
hypothetical protein	CAAAATTGTTAACTGATAACTGATAATTGAAAAAAATGGCTATTCTGCGTTTAATG	00155_2445	hypothetical protein	
succinate-semialdehyde dehydrogenase [NAD(P)+]	TGATGACT~77nt~GGTTTCTTGCATAACTGATAATTGATAACTGATG	01101_2504	putative lipoprotein	
PAS domain S-box	TAATGATTAGCGTGTTAGAAATACCCTTTTTTTGAAAAAAGGGTATTTCTTTCTTTAACTGATAACTAATAACTGATATG	00701_1072	conserved hypothetical protein	
hypothetical protein	(>100 bp to next ORF) TCCGTTATTTTGTTTTGGGAACGAAGAAATATTTTTCTCTAACTGAAAATCTGATAACTGACAATG	00391_1528	response regulator receiver domain protein	
conserved hypothetical protein COG4198	CATTTATAACTCCTGGTGAATTTTGATTTTTGAATTAATAATTAATAATTGATAACTGATAACTGGTAACTGATAATATG	01192_0218	hypothetical protein	
S-adenosylmethionine:tRNA ribosyltransferase-isomerase QueA	CATAAGTAGGTAAA~82nt~ATTCTGCTAACTGATAACTGTTAACTGTTAACTGATAGCC	00153_2340	tRNA-Leu-CAA	
prepilin-type N-terminal cleavage/methylation domain protein	TTAATGGTTTAACTGATAATTAATAACTAATAACTGAAAATG	00936_0896	prepilin-type N-terminal cleavage/methylation domain protein	

Upstream ORF	Repeat region sequence	Downstream ORF		Downstream COG category
signal peptidase I	TAATTCAATTATCAATTATCAGTTA CCAG~29nt~TTTGAA TGATAACTGATAACTGA TAACAAATAACAAATAACGAATAACTAAATATTATG	00917_2246	hypothetical protein	
hypothetical protein DUF4384	CATGTTATGGATATGTCATTCAATAGATTAAAAGGCATGACGCAGGCGCGTTAGAAGCATAA ATAACTGATAACTGATAAC GAA TAACTGAAATG	00855_2690	conserved domain protein	
hypothetical protein	CATTCAGTATAACCACTGAATAATTGATAACTGATAACTGA TCACTGATAACTGATATG	00396_2166	HAMP domain protein	
aspartyl/glutamyl-tRNA amidotransferase subunit C GatC	TAGTTAGTGTT~175nt~GTGAATGCTTAACTGA TAACTTA ATAACTGATAACTGA TA GCTAAATTATG	00150_0829	aspartyl/glutamyl-tRNA amidotransferase subunit A domain protein	
tRNA-Pro-TGG	CCGTTGAATC~128nt~TGGTTAATAGTTGATGATAACTGA TAACTGGTAACTGATAACTGATATG	00822_0370	hypothetical protein	
hypothetical protein	TAAAGTATTTATAAACCAAGTTTTTCAAAAAACTTGGTTTCTTTAGATTCTAAGA TAACTGATAACTGATAACTGATATG	01113_1161	DNA gyrase, B subunit	
peptidase, M48 family	(>100 bp to next ORF) GAACTTTTAT ATAACTGATAACTGATAACTGATTTATG	00244_2475	hypothetical protein	
methyltransferase, FkbM family	(>100 bp to next ORF) AAGGTATATATTTTTACCAGATTATAACTGATAACTGATAACTGAAATGATG	01087_4783	hypothetical protein	
LysM domain protein	(>100 bp to next ORF) ACGAAAGTTACTATGGACGCAT TGATAACTGATAACTGATAACTGAAAACTGTCATG	01092_1315	LysM domain protein	
Uncharacterized protein conserved in bacteria DUF2333	TAAATTCGAATTTCAGAAATACGATTTCTTGAAAAAATCGTATTTCTTATAAAATTTTACACTAAATTTA TAACTGATAACTGATAACTGATAACTAATAATATG	00938_0737	glycosyltransferase, group 2 family protein	
hypothetical protein	(>100 bp to next ORF)CGTGCAGTATACACTGATAACTGATAACTGATAACTGATA TGGACGACGTTAAATTATTGCTATATGTG	01054_0111	hypothetical protein DUF4276	(none assigned)
CheR methyltransferase, SAM binding domain protein	AAGAATTTATCGCCGATTAAAC ACTGATAACTGATAACTGATAACTGAAA ATG	00806_2998	protein-glutamate methylesterase CheB	
PAS domain S-box	GGCTAAAAATTTTGAAATGTCTAAACTTAAAAAATTGATAACTGATAACTGATAACTGATTATG	01232_0432	response regulator receiver domain protein	
putative flagellar protein FliS	(>100 bp to next ORF)AAAATTATCTTTATATACGATTATAACTGATAACTGATAACTGATAATTGATAACTGAAATAAATAATG	00163_0993	Hpt domain protein	
V-type H(+)-translocating pyrophosphatase	(>100 bp to next ORF) TTTAATGAGCTTACTGATAACTGA GATAACTGATAACTGATAACTGAAATG	00848_4301	conserved domain protein, ABC transporter type 1, transmembrane domain	
hypothetical protein	CAACCAT TGATAACTGATAACTGATAACTGATAacTGAtaTg	00059_1188	Domain of Unknown Function (DUF349)	
S-adenosylmethionine-dependent methyltransferase, YraL family	TGATAACTGATAACTGATAACTGATAACTGACATG	00398_4584	histidinol-phosphate aminotransferase domain protein	
N-acetyl-gamma-glutamyl-phosphate reductase	TAATGGGAAATTGTGA~77nt~GATAATTATTTATGTGATAACTGATAACTGATAACTGATAACTGAACAATG	00369_1642	hypothetical protein	
putative glycoprotease GCP	(>100 bp to next ORF) GTTGTTTAGAATATATTTCTAACTGATAACTGATAACTGATAACTGATAACTGGTAGCCGAAATG	00162_0484	conserved hypothetical protein	
multiheme cytochrome	(>100 bp to next ORF)TACTATAATTGGGTTAAATTTTATCACGCTCCACTGATAACTGATAACTGATAACTGATAACTGATAAGAATTGGATAAAATAATG	01191_1502	hypothetical protein	
glutamate--cysteine ligase GshA	(>100 bp to next ORF) GAATTGGATATCAAAAATGAATTTTAACTGATAACTGATAACTGATAACTGATAACTGATAACTGAAATG	00163_0989	response regulator receiver domain protein	
DNA repair protein RadC	(>100 bp to next ORF)CAATA ATAACTGA CAAAAAATTTTTA~35nt~TGG TAACTGA TAAC TAACTGA TAACTGGTAATTGATAACTGG TAACTGA TAAAT TGATAACTGATAACTGAAAAAGCCA	00285_1246	tRNA-Val-CAC	

(B)

Upstream ORF	Repeat region sequence	Downstream ORF		Downstream COG category
hypothetical protein 00212_4968	TAA~423nt~ATTAAATAACTGATAACTGATAACTGAtT		(end of contig)	
hypothetical protein 00766_5221	CATCGAACCTAACGGTTCAATTATTTGTCTGAAAAACGGATAACTGATAACTGATAACTGAAATGATGTTAAACT		(end of contig)	
hypothetical protein 00832_2874	TAGGGTAATAT~56nt~ATAAGCAGATAACTGATAACTGATTGCTAAAAATTTTTTtGGTTTTA~468nt~TGGTTTAGTTGTCGAATAACTGATAACTGATTATTGATTACTGAAAGC		(end of contig)	
conserved hypothetical protein (putative transposase or invertase) 00950_4759	TAGGGTAAATA~54nt~CATAAACTGATAACTGATAACTGATTATTG~442nt~TTGACCTGGCAGGTCTGGTTTAGTTGTCGAATAACTGATAACTGATTATTGATTACTGAAAACTT		(end of contig)	
conserved hypothetical protein 01350_4606	TAACGTCAA~50nt~TTGCAATAACTGATAACTGATTAGAAATT~186nt~TCTGATAATTAATAACTGAGGGAAAAATCGAAGTGGATGAACTGATAGAACTTGAAAATGAGGAATGGG		(end of contig)	
hypothetical protein 01356_5323	TAACGTCAAAGAAATCCTATTTTTCTAAAAAATCGGCTTTCTCAGTTCAGGGCAATCTTTTACAATAACTGATAACTGA		(end of contig)	

(C)

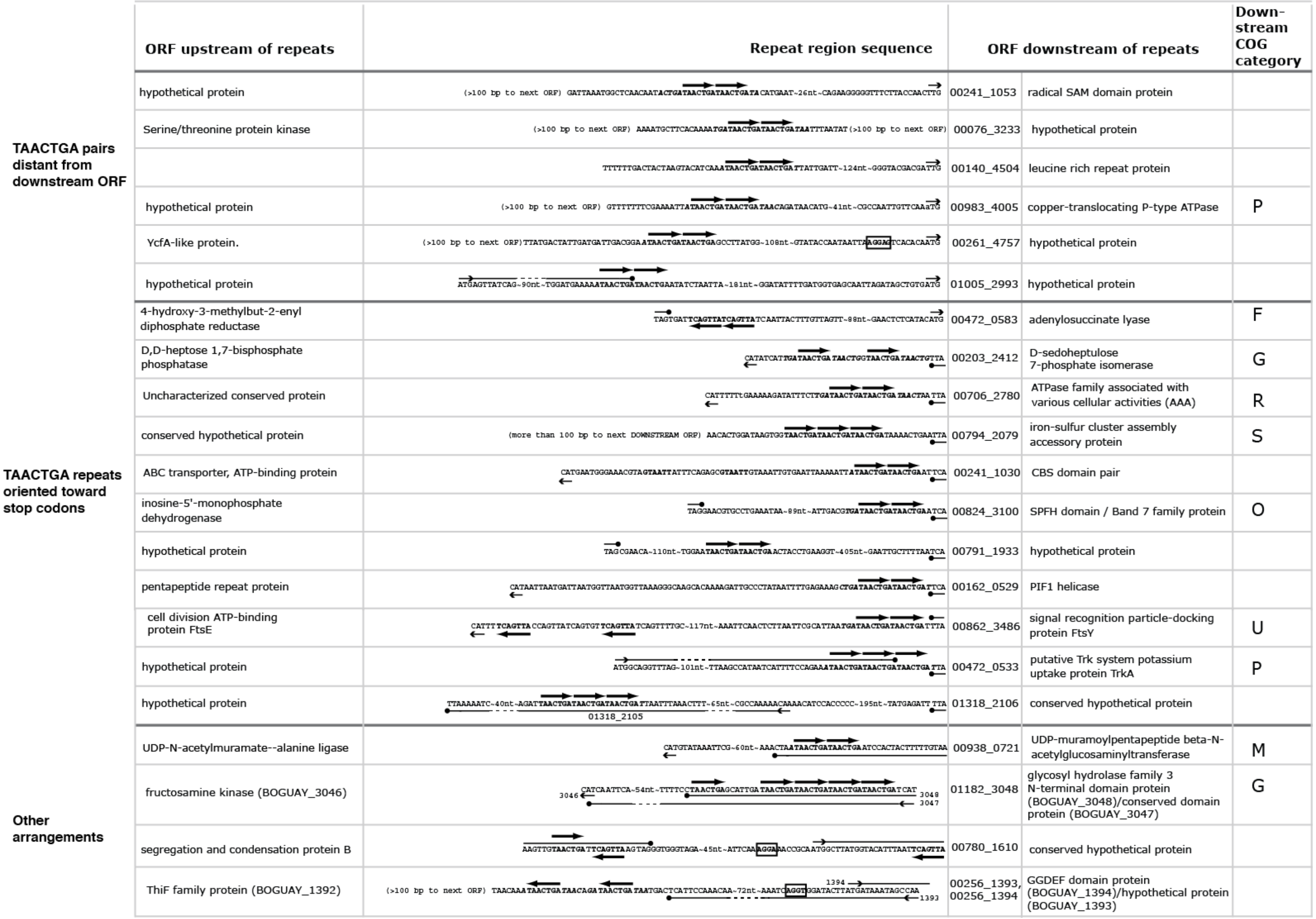
ORF upstream of repeats
Repeat region sequence
ORF downstream of repeats
Down-stream COG category
TAACTGA pairs distant from downstream ORF
hypothetical protein
00241_1053
radical SAM domain protein
Serine/threonine protein kinase
00076_3233
hypothetical protein
00140_4504
leucine rich repeat protein
hypothetical protein
00983_4005
copper-translocating P-type ATPase
P
YcfA-like protein.
00261_4757
hypothetical protein
hypothetical protein
01005_2993
hypothetical protein
TAACTGA repeats oriented toward stop codons
4-hydroxy-3-methylbut-2-enyl diphosphate reductase
00472_0583
adenylosuccinate lyase
F
D,D-heptose 1,7-bisphosphate phosphatase
00203_2412
D-sedoheptulose 7-phosphate isomerase
G
Uncharacterized conserved protein
00706_2780
ATPase family associated with various cellular activities (AAA)
R
conserved hypothetical protein
00794_2079
iron-sulfur cluster assembly accessory protein
S
ABC transporter, ATP-binding protein
00241_1030
CBS domain pair
inosine-5'-monophosphate dehydrogenase
00824_3100
SPFH domain / Band 7 family protein
O
hypothetical protein
00791_1933
hypothetical protein
pentapeptide repeat protein
00162_0529
PIF1 helicase
cell division ATP-binding protein FtsE
00862_3486
signal recognition particle-docking protein FtsY
U
hypothetical protein
00472_0533
putative Trk system potassium uptake protein TrkA
P
hypothetical protein
01318_2105
01318_2106
conserved hypothetical protein
Other arrangements
UDP-N-acetylmuramate--alanine ligase
00938_0721
UDP-muramoylpentapeptide beta-N-acetylglucosaminyltransferase
M
fructosamine kinase (BOGUAY_3046)
3046
3048
3047
01182_3048
glycosyl hydrolase family 3 N-terminal domain protein (BOGUAY_3048)/conserved domain protein (BOGUAY_3047)
G
segregation and condensation protein B
00780_1610
conserved hypothetical protein
ThiF family protein (BOGUAY_1392)
1394
1393
00256_1393, 00256_1394
GGDEF domain protein (BOGUAY_1394)/hypothetical protein (BOGUAY_1393)

98

Supplemental Table 2. TAACTGA repeat distribution in *Beggiatoaceae* and other genomes. Only perfect direct repeats (no split sets) were counted as doublets etc. here. Within each taxonomic group, strains are in order of decreasing number of doublets.

**Strain**	**Total TAACTGA**	**Repeats per set**														
		**1**	**2**	**3**	**4**	**5**	**6**	**7**	**8**	**9**	**10**	**11**	**13**	**14**	**38**	**41**
***Beggiatoaceae* (GAMMAPROTEOBACTERIA)**																
Cand. “Thiomargarita nelsonii”	1239	764	112	68	9	1	-	-	-	-	-	-	-	-	-	-
**Orange Guaymas “Maribeggiatoa”**	**1357**	**1006**	**68**	**40**	**19**	**1**	**1**	-	-	-	-	-	-	-	-	-
Thioploca ingrica	1398	1345	20	3	1	-	-	-	-	-	-	-	-	-	-	-
Beggiatoa alba	625	625	-	-	-	-	-	-	-	-	-	-	-	-	-	-
***Chromatiaceae* (GAMMAPROTEOBACTERIA)**																
Thiocystis violascens DSM 198	129	123	-	-	-	-	1	-	-	-	-	-	-	-	-	-
**CYANOBACTERIA**																
Chroococcidiopsis thermalis PCC 7203	1786	1389	106	44	13	1	1	-	-	-	-	-	-	-	-	-
Microcystis aeruginosa NIES-843	2371	1295	97	39	61	25	16	6	7	4	5	1	2	-	1	1
Calothrix PCC 7507	2154	1533	85	53	33	11	5	5	1	1	1	-	1	-	-	-
“Nostoc azollae” 0708	1542	1324	41	11	7	4	1	1	1	-	2	-	-	1	-	-
Stanieria cyanosphaera PCC 7437	1726	1588	37	14	-	3	-	1	-	-	-	-	-	-	-	-
Calothrix PCC 6303	1617	1530	34	5	1	-	-	-	-	-	-	-	-	-	-	-
Nodularia spumigena CCY9414	1360	1100	32	26	19	6	2	-	-	-	-	-	-	-	-	-
Rivularia sp. PCC 7116	1813	1738	29	3	2	-	-	-	-	-	-	-	-	-	-	-
Pleurocapsa sp. PCC 7327	770	739	9	3	1	-	-	-	-	-	-	-	-	-	-	-
Cyanothece PCC 8801	1020	996	9	2	-	-	-	-	-	-	-	-	-	-	-	-
Halothece sp. PCC 7418	891	769	9	2	-	-	-	-	-	-	-	-	-	-	-	-
Cyanothece PCC 8802	1000	970	5	4	2	-	-	-	-	-	-	-	-	-	-	-
Nostoc sp. PCC 7107	1300	1280	5	2	1	-	-	-	-	-	-	-	-	-	-	-
Crinalium episammum PCC 9333	1449	1439	2	2	-	-	-	-	-	-	-	-	-	-	-	-
Cyanothece PCC 7424	1503	1494	1	1	1	-	-	-	-	-	-	-	-	-	-	-
Cyanothece PCC 7822	1840	1837	-	1	-	-	-	-	-	-	-	-	-	-	-	-
**BACTEROIDETES**																
Flexibacter litoralis DSM 6794	839	798	11	1	-	-	-	1	-	1	-	-	-	-	-	-
Elizabethkingia anophelis NUHP1	723	713	4	-	1	-	-	-	-	-	-	-	-	-	-	-
Paludibacter propionicigenes WB4	502	496	1	-	1	-	-	-	-	-	-	-	-	-	-	-
Gramella forsetii KT0803	590	556	-	-	-	-	-	-	-	-	-	-	1	1	-	-
Aequorivita sublithincola DSM 14238	574	562	-	1	-	-	-	-	-	1	-	-	-	-	-	-

Supplemental Table 3. Sample of TAACTGA repeats within the *Microcystis aeruginosa* NIES-843 genome. Sequences were selected at random from among those identified by a blastn GenBank nr search with 7 direct TAACTGA repeats. "Forward" repeats are in boldface, and reverse repeats in boldface red.

**Upstream ORF**		**Intergenic region**		**Downstream ORF**	
**Locus tag**	**Description**	**Position**	**Sequence with flanking start and stop codons**	**Locus tag**	**Description**
**Forward (relative to start codon of downstream ORF)**					
MAE_03920	hypothetical protein	353997..354052 (reverse complement here)	<CATCTGATAACTTTTAACGCCTACTTACTTAACTGA**TAACTGATAACTGATAACTGAA**AATG>	MAE_03910	cobyrinic acid a,c-diamide synthase
MAE_07750	hypothetical protein	670646..670882	>TAAATTTAACGGTCGGAAAACAGGAGATCGGAAGTGGGAATTATGAATTATCAATTATGAAGTGGGGTGGTGGGGAAGTGGGGAAGTGGGGAGACCACTTCGTGCGCGTTGCGGGGGGAGAATAAAAGCGTTCGACTGCTCACTTAAAACTGATAACTGCTCACTTAAAACTTAAAACTTAAAACTGA**TAACTGA**TAACTTAAAACTGA**TAACTGATAACTGA**TAACTAA**TAACTGA**ATTATG>	MAE_07760	hypothetical protein
MAE_07270	glutathione synthase (EC 6.3.2.3)	636108..636264 (reverse complement here)	>TGACACACAATATTTAAGGCTCTGTATTAAAATATCCTTCAGGTAGAACCTGAACAAAGGGTTCCCAGGTTCTACCTGGGAACGAGATAAGAACTCAAATCTGA**TAACTGATAACTGA**TAACTTACCCACTGA**TAACTGATAACTGA**TAACTGTAAAAAAATG>	MAE_07260	hypothetical protein
MAE_02870	DNA-(apurinic or apyrimidinic site) lyase (EC 4.2.99.18)/endonuclease III (EC 3.2.2.-)	250091..250246	>TAAACAGTAAACTCACACCTGA**TAACTGA**ATCACTGA**TAACTGA**TAACTGTAAACTTAAAACTCACACCTGATAACTGG**TAACTGA**ATCACTGA**TAACTGATAACTGATAACTGA**TGTGGGGGGCTATAATGGGAAAAAATAGCTCATCCATCAATCTTATG>	MAE_02880	putative peptidase
MAE_00910	hypothetical protein	71125..71387	>TGATCAACCTCGTTTTGTTTTAAGGTAGGCTAAGGCCTGCCTTACTTTTTATCAGTGATCAGTAACCAGTAAGCAGAAAATTGCCATTTAATACCAATCAGCGCTCAGACTGTCCACGGAAAACTCAAATCTGATCACTGG**TAACTGATAACTGATAACTGA**TCTGATCCCCTAGCGTGATAAGCTGTGTATTGGCTAATTTTACAAAAAATTAAGACTTTCCCCTTGTGGGAGTTATCAGCTAAAACCCAGAGTAGACATAAACAATG>	MAE_00920	ATP synthase F1 subcomplex beta subunit
MAE_01930	hypothetical protein	165816..166136	<CATAGTTAAAAAATTCAGTCAATGAAATGTACTTATTGATAAGCTGTTGACTCTTGAATGATAGCTTTCAGCCATGAGCTTTCCACTGA**TAACTGATAACTGATAACTGA**TTACTGATTACTGATTAACTTGACTGCCGTGACTGCGAGGATCGGTTTGACTAGCGCCCGCAAGGATATTAAAATCAGAAGCCTGTCCCGTAGTGGTGGCATAGGGTAGGGTAGAATTGGTTAAAAGTGCTTCTAAATTAGCGGTTAAAATTGATTTTGGCTGTTCTCCGATCGCTTGGGCAATCGGTTGTCCTTGATTATTTAAAAAGACAAAATG>	MAE_01940	hypothetical protein

112

Reverse (relative to start codon of downstream ORF)

MAE_01210	hypothetical protein	99134..99404 (Reverse complement shown here)	>TAGAAAGGCATTTAGCGATTTTTGTCAATTATTTTTGATCTAGAGCGAACTAAT<br>CAATTAAGTCTCTTACCAGATAAGGATTTAGTCGATTTATGACCCCCTATCGAAC<br>CATACCAAGTAACGAAGAACCATTTA**TCAGTTA**TCAGTGA**TCAGTTATCAGTTA**T<br>CAGATGTGAGTTTTCAGTTCACTGATTACTGTTCACTGAAAATGCTTCCCACTTC<br>CCAATTCATAATTCATAATTCATATTACTGTCTGCTATCTCCTTTTTACCTTACC<br>ATG>	MAE_01200	neutral amino acid ABC transporter ATP-binding protein
113					
MAE_00300	neutral amino acid ABC transporter membrane protein	24891..25171	>TAATT**TCAGTTATCAGTTATCAGTTATCAGTTATCAGTTATCAGTTATCAGTTA**<br>**TCAGTTATCAGTTATCAGTTA**TCAGTGATTAGATTTGAAGATTAAGTGAACAGTA<br>TTAAATAGCAGTTTTCTAAGGACTTAAGCAAAATTAATTACACATTTAAATAATC<br>CTATTGCAAGAGTGCCTATTGCCTGTCTCAACTAAGAAATTTATTTCCCACGACT<br>ACTTACTGTCTTTTCACTGATTACTGTTTAATGTTTACTGA**TAACTGA**TGTTTAC<br>CTGATCAATTATG>	MAE_00310	neutral amino acid ABC transporter ATP-binding protein
MAE_02000	hypothetical protein	171470..171517 (reverse complement shown here)	>TAACAGTTA**TCAGTTATCAGTTA**TCAGTTGTTTCTAACCATCTACTATAATG>	MAE_01990	hypothetical protein
MAE_03510	hypothetical protein	314995..315226 (reverse complement shown here)	>TTAATCGGTGCATTACGCTATCGCGCTTCACAGCCTACGAGTAAGCAAACAACT<br>CAAATGAACCAAACAACCGCCAATTATGATGAACCCTGGAAAGAAGCATTAAGCG<br>AGTATTTTGAAGCGTTTTTATACTTCTTTTTTCCTGAAGTTCACCAACTAA**TCAG**<br>**TTATCAGTTATCAGTTATCAGTTA**TCAGTGACTAATTGGAAACAAGTAGCAATCC<br>CTTTGCTATAATAGTCATG>	MAE_03500	hypothetical protein
MAE_10110	putative methyltransferase	871106..871421 (reverse complement shown here)	>TAAAAAAGCAGCGTCGAGACAAGTTCGATCCC**TCAGTTATCAGTTA**CCAGTTAC<br>CAGTTA**TCAGTTA**CCAGTTA**TCAGTTATCAGTTA**CCAGTTACCAGTTA**TCAGTTA**<br>**TCAGTTA**CCAGTTA**TCAGTTATCAGTTATCAGTTATCAGTTATCAGTTATCAGTT**<br>**ATCAGTTATCAGTTATCATTGA**TTAGTTATTGGTCACTGTTTACTGTTTACTAAT<br>TACTGTTTACCGTTTACTGTTCAATGTTTACCGTTCACTGATTACTGTTTACTGA<br>TTACTGTTTACTGATTACTGTTTACTGATTACTGTTTACTCTCCCATG>	MAE_10100	hypothetical protein
MAE_00820	cobalt transport system permease protein CbiQ homolog	61945..62434	>TAGCAGTTA**TCAGTTATCAGTTATCAGTTA**TCAGATTTGAGTTTTAAGTGTGCA<br>GTATTAAATAGAAGTTTCCTACTGTCTTTGCACTGATTACTGATTACTGTTTACT<br>GATTACTGTTTACTGTTCACAGCAAAAAACTCCCCACACCCTACACCCAGCCTAG<br>ACTTTAACTGCGTTGTCACCCCTTCAGGGTGCGGATGTTGACGGCAATGTTGTGG<br>AATTTATAACCGAGGAACCCTTCGCGCCTCCGGGAGGAGTAGTAATTAGGGTCTG<br>CTGAATAAATCTAAAAACTTTGTTGGATAAGACTTTTGGACTTTTTTCCCCTCAA<br>AAAGTGCCAGCCATTGCGGGGATCGGGGGGAAATTCAGGGACTTTTTCCCTGAAA<br>ATTAGGTAGTTGACCACCTGAAAATCGATAAAACCCCACACCCCACACCCCACAC<br>CCCACACCCTGCCCCCAGGAAAAGCTTTTTGCCGCAAACCCTAATTATCCCTCTG<br>TG>	MAE_00830	hypothetical protein

114

MAE_03520	hypothetical protein	316442..316841	>TAAA**TCAGTTATCAGTTATCAGTTA**TCAGTGATCAGTGATCGGATCTAAGTAGC<br>TGGTTATAATTAAATTAAAAATGGATTTTGCCTTTAATCCCCCCTTGATAAGGGG<br>GGTATCTGATAATTTTTAACGCCTACCTACTTAAGTTTTAAGTTAGTTAGTTAGT<br>TAGTTATAGCACTTTTCACGTTACTGAGGTATCGACAAGTTTTGCCAACAAAGGG<br>TTTAATATCCTTGCCCATACCTCATTCTGATGAAAACTGCTATATTTATCTTCAT<br>CTTTATCCCTGTTTACTGTTTACTGA**TAACTGA**TCACTGATCACTGATCACTGAT<br>TACTGACAATTCTCCTTCTCCGCCGCGTTAACCATAGTCGATAATAATAAAAGAC<br>AGATCGATCGCTTGCGCTCATG>	MAE_03530	molecular chaperone
MAE_06690	tRNA(Ile)-lysidine synthase	587484..587825 (reverse complement)	>TGA**TCAGTTATCAGTTATCAGTTA**TCAGATTACAGTTTTAAGTGGACAGTGTTA<br>GGTGAAAACTTCGCTACTTTCTTTTCACTGATTAAGTGGGTGGGTGGAATTAAAT<br>ATAAGATGAACGTAGGTTGGGTTGAAGCATGAAACCCAACACCCGCTCATGTTAC<br>GCTACCGCTAACCCATCCTACAAATAATTGTGCCTCCCTACTTACTGCTTACTGT<br>TTACTGTTCACTGAAAAACCCCATCTCCCCATTCTAGTTATCTCTAGCTAACTTT<br>TACAGCAATTAGTTTAAGGAGAAACTAAAATAGTAATACAATTAAACTAAAAATA<br>AAATTGGCGGGGCATAATG>	MAE_06680	putative DNA helicase
**Between stop codons**					
MAE_00040	2-isopropylmalate synthase (EC 2.3.3.13)	3655..3757	>TAGGGAACAGTTA**TCAGTTATCAGTTATCAGTTATCAGTTA**TCAGATTTTTTAT<br>CCCCACACCCTACACCCCACTTCCTGCTCCCCTTTCTGGGATTGAATGTTTCCTA<br><	MAE_00050	hypothetical protein
MAE_01300	polyphosphate kinase	110501..110759	>TAGGAACAGTTA**TCAGTTATCAGTTATCAGTTATCAGTTA**TCAGTGGGTAAGTT<br>GTCAGTGATCAGATGTGATACTAAATCTGGTTATTAAAAACTGATTATCTATTGC<br>TCCTTTTGCCTATTGCCTATTGCCTCTCCTGATATGTAGCCTATACTCAACGGAT<br>TTAGTATGACTAACTGGTTGAGAGGTTAAACCATCTCAAAAGCTGAGAGCAAGCA<br>TCTTGATCTGTATTTTCTCAACCAGTCAAATCAAGCAAGAATCCTA<	MAE_01310	flavoprotein
MAE_02120	hypothetical protein	182025..182314	>TAATTAGGGTTTACTGAA**TCAGTTATCAGTTA**TCAGTAATCAGTAA**TCAGTTAT**<br>**CAGTTA**AGTAGGTAGGCGTTAAAAATTATCAGATCCCCCCGCCTATCGGCACCCC<br>CCTTATCAAGGGGGGCAGGGGGGATCGAACCTAAAATCGATTTTAAATTTAATTA<br>TAACCAGTTACTTATCAGTGATCAGATGTGAGTTTTCAGTTTACATTTTTATCAG<br>TAATCAGTAATCAGTTCACTGTTTACTGATTACTGTTCACTGATTACTGTTTACT<br>GAAAATACACCCCACTTCCCTA<	MAE_02130	5-(carboxyamino)imidazole ribonucleotide synthase
MAE_02170	neutral amino acid ABC transporter membrane protein	187374..187601	>TAACAGTTA**TCAGTTATCAGTTATCAGTTATCAGTTA**TCAGATGTAAGTTTTAA<br>GTTTGAAGTTTTAAGTTATTTATAGCGCTTTTCACGTTACTGAGGTATCGACAAG<br>TTTTGCCAACAAAGGACTTAAGCCCCTTGCCCATGCCTCATTCTGATGAAAACTG<br>CTATATTTACTGTTTACTGATTACTGTTTACTGATTACTGTTTACTGATTACTGA<br>AAAGCCCAATCTATT<	MAE_02180	hypothetical protein
MAE_02480	DNA replication and repair protein RecF	210466..210811	>TAGCCTATAGCCACCAGCCTTCAGCCATAT**TCAGTTATCAGTTA**TCAGGTTTTT<br>CTCCCTATCTTTCCACTCTCTAGTCACCTCAGCAGTTTAACCCTAAACTAAAGCT<br>ACTCATTCAGTAAATAGCTGCGATATCTCGGTAGTTTGCTCTAGAAAATTTCTCA<br>TATCTTCTGGTTTAATTGTTTGGTTTAATTTATAAATTACTAACCATTCACCACC<br>AGCTTGCAAATACCAACCATGGTTCTGTCCAAAAAATTGTCAGGTGTCAAAATTA<br>AAGATTTCTCGGATAGTAGATTCTTACCATAATACGATCTCAAGCTTATGAACCT<br>TTATTTACAGACCATTGCTGTCA<	MAE_02490	hypothetical protein

MAE_03940	pyruvate kinase (EC 2.7.1.40)	358054..358154	TAA>GCAGTT**TCAGTTATCAGTTA**TCAGTCGGAACTGTGTGCTGATGGGAAGTGA<br>GGGAGTGAGCAGTTAATCGTACTATACAAAAGCGGATTTGGGAGTAGCGGATT<	MAE_03950	hypothetical protein
MAE_08080	spermine synthase	701721..701789	>TAA**TCAGTTA**TCAGTAATCAGTAATCAGTAA**TCAGTTATCAGTTA**TCAGCTATC<br>AGTAAATAAGGGTTATAACTA<	MAE_08090	putative exonuclease, RecJ-like
MAE_10360	sodium/solute symporter	890402..890961	>TAGGCAGTTA**TCAGTTATCAGTTATCAGTTATCAGTTA**TCAGTTTTAAGTTTTC<br>AGTTTTCAGTTT**TCAGTTA**TCAGTTTTCAGTTTTCAGTTTTCAGATTTGAGTTTT<br>AAGTGTGCAGTATGGATTAGGTGAGCCTCATCAAATGCCAGTTTTCTACTGTCTT<br>TTCACTGATTACTGTTTACTGATTACTGTTTACTGAAAATAGCAGTTTCCTACTG<br>TCTTTTTACTGATTACTGATTACTGATTACTGATTACTGATTACTGTTTACTGAA<br>AATAGCAGTTTTCTACTGTCTTTTCATTGTTTACTGTTTACTGATCACTGAATTA<br>GGAGAGATCCCTTGACTTTTGCGGCGGCTTGGGTTATGATTTTCTTATAATGGGA<br>CTGTTGTGTTTATGGTTAAGTGGTTATATTTCCATTATTATTAAACTCTTTTAAA<br>AATACTGTCCCAAATCTGCCAAAAAAAGCAAGCAAAATTTTGCCGTCAATTGTTA<br>AGTTTTGTTAAGAAAAATAGCAAGAGCGGCAAGGAATAGAAATCCCTTACCGTTG<br>ACTTTGGCCGGACTCA<	MAE_10370	cyanate lyase

Supplemental Table 4. TAACTGA repeats in *Cyanothece* strains PCC 8801, 8802, and 7424. TAACTGA units are in boldface, with those in "reverse" orientation in pink. Putative start and stop codons and Shine-Dalgarno sequences are underlined. Gaps were added to bring regions of obvious similarity into alignment. The table includes all repeat units identified in PCC 8801, 8802, and 7424; PCC 7822 sequences were identified as having the best blastp hits to downstream PCC 7424 ORFs.

Strain	(Upstream) locus tag	(Upstream) ORF	Downstream locus tag	Downstream ORF	Sequence
**8801 and 8802, inserts in matching positions**					
8801	PCC8801_0895	peptidase M16 domain protein	PCC8801_0896	fructosamine kinase	TAGGGTAGGGGAGGGTGGGGAGAGTGGTAAGAGTCGGAAGAGAGGGGAGATACTAACTTCTGACTCCTGACTTCTGCCTATTGCCTATTTACTAA**TAACTGATAACTGA**AAGATG
8802	Cyan8802_0921		Cyan8802_0922		TAGGGTAGGGGAGGGTGGGGAGAGTGGTAAGAGTCGGAAGAGAGGGGAGATACTAACTTCTGACTCCTGACTTCTGCCTATTGCCTATTTACTAA**TAACTGATAACTGA**AAGATG
8801	PCC8801_2652	signal peptidase I	PCC8801_2653	1-(5-phosphoribosyl)-5-amino-4-imidazole-carboxylate (AIR) carboxylase (circadian clock modulator)	TAACGCTGAATTACTGA**TAACTGATAACTGATAACTGA**TTACTGAAATG
8802	Cyan8802_3452		Cyan8802_3451		TAACGCTGAATTACTGA**TAACTGATAACTGATAACTGA**TTACTGAAATG
8801	PCC8801_2677	ribose-phosphate pyrophosphokinase	PCC8801_2676	UPF0047	TAGCTATCTTTGAAT~148nt~GATGATGTTATCAGTCGTTATCCATTACTGA**TAACTGATAACTGA**TAATTTATTACTGAAATG
8802	Cyan8802_3427		Cyan8802_3428		TAGCTATCTTTGAAT~148nt~GATGATGTTATCAGTCGTTATCCATTACTGA**TAACTGATAACTGA**TAATTTATTACTGAAATG
8801	PCC8801_3489	Indole-3-glycerol-phosphate synthase	PCC8801_3488	hypothetical protein	TAACTTGTGAGAGGG~204nt~TCTACTTCCCTTACTGA**TAACTGATAACTGA**CAACTGATAACTAATATG
8802	Cyan8802_2627		Cyan8802_2628		TAACTTGTGAGAGGG~204nt~TCTACTTCCCTTACTGA**TAACTGATAACTGA**CAACTGATAACTAATATG
8801	PCC8801_4057	Hypothet-ical protein	PCC8801_4058	hypothetical protein	<CATTTTTTCTGACCT~97nt~TTATTTAAGTCGATTTCTTGA**TAACTGATAACTGA**TCACTGATG>
8802	Cyan8802_4096		Cyan8802_4097		<CATTTTTTCTGACCT~97nt~TTATTTAAGTCGATTTCTTGA**TAACTGA**TCACTGATCACTGATG>
8801	PCC8801_2460	hypothetical protein	PCC8801_2459	band 7 protein	TAGGAGAGAAGAGGAGACTAGAAGAAAATATAAACGATGGATTATTGTTTGCTGA**TAACTGA**TTACTGATTACTGA**TAACTGATAACTGA**TTACTGAATCCTATG
8802	Cyan8802_3649		Cyan8802_3650		TAGGAGAGAAGAGGAGACTAGAAGAAAATATAAACGATGGATTATTGTTTGCTGA**TAACTGA--------------TAACTGATAACTGATAACTGA**ATCCTATG

8801	PCC8801_3657	ATP synthase F1, beta subunit	PCC8801_3656	ATP synthase F1, epsilon subunit	TAATCTCTGCCAATAGGTTGTCAGTTAACAGTCATTTAGAGGCATCTCTTCCAATTTACTGTTAACTGGCCTGA**TAACTGATAACTGATAACTGA**-------TAACTAA**TAACTGA**TATG
8802	Cyan8802_3711		Cyan8802_3710		TAATCTCTGCCAATAGGTTGTCAGTTAACAGTCATTTAGAGGCATCTCTTCCAATTTACTGTTAACTGGCCTGA**TAACTGATAACTGATAACTGATAACTGA**TAACTAA**TAACTGA**TATG
8801	PCC8801_0918	hypothetical protein, yfbK Ca-activated chloride channel homolog	PCC8801_0919	hypothetical protein, tubulin-like	TAAA-TTTTCTATCATCTAAATTAGTTGTTGACTGGGATATGAGGTAACGGGGAGATAAT**TAACTGATAACTGA**AAGGAGGGAATGATG
8802	Cyan8802_0945		Cyan8802_0946		TTAAGTTTTCTATCACCTAAATTAGTTGTTGACTGGGATATGAGGTAACGAGGAGATAAT**TAACTGATAACTGA**AAGGAGGGAATGATG
8801	PCC8801_1611	twitching motility protein	PCC8801_1612	type II secretion system protein	TAATGACAGTTATCAA--------------TTA**TCAGTTATCAGTTA**TCAGTGATTAGCTTTGAGTCTTAAATTGCTTACTGTTAACTATTCACTATTCACTATTCACTAT-------AAAACGATG
8802	Cyan8802_1637		Cyan8802_1638		TAATGACAGTAATCAGTAATCAGTAA**TCAGTTATCAGTTATCAGTTA**TCAGTGATTAGCTTTGAGTTTTAAATTGTTTACTGTTAACTATTCACTATTCACTATTCACTATTCACTATAAAACGATG
**8801 and 8802, unique inserts**					
8802	Cyan8802_1813	NifH nitrogenase iron protein	Cyan8802_1814	nitrogenase molybdenum-iron protein alpha chain	TAATGTAAGGGCTTAATTACCTTAAGCCCTGCAATTGGGAAATGGGGAGAGTAACAGTAAACAGTAAATACTGA**TAACTGATAACTGA**TAACTGTTTTAACCTCCCTTTACCCCCATCCTCATCAAAATTTCCTACTCGTCATTATTGACGAAGGCGTGCGTCCGATCCTAATCGCCAATTCATCACTGAGGAACACTATG
8801 (plasmid)	PCC8801_4541	transcriptional regulator	PCC8801_4540	Relaxase/mobilization nuclease family protein	TGAATTATT~39nt~CTTT**TAACTGATAACTGA**TAACTGTTAAACGGCTCTCATCTATAATCTTT~82nt~CCAAACTAAGTTAAGCCAATG
8802 (plasmid)	Cyan8802_4609	pseudogene	Cyan8802_4610	GUN4 domain protein	TTACCAATCCCTTC~184nt~TTGTGAACTGTACT**TAACTGATAACTGATAACTGA**TAACGGTG~74nt~TAATGGCGATATATTA<
8801	Within: PCC8801_2480	DUF323; encodes aa 432-441 (SVNSYQLSVI)			ATG~1291 nt~ **TCA GTT A**AC AGT TA**T CAG TTA TCA GTT A**TC ~429 nt~TAG

PCC 7424 and closest relatives					
7424	PCC7424_2144	phage shock protein A, PspA	PCC7424_2143	Thioredoxin domain protein	TAGTCATCAATCAATCATAATCAGTGATACAGTTATCGGTAAATAGGGAACAGTTATCACCGATAATGATTTACTGAGAA------------------------CTAA-**TAACTGATAACTGA**CGACTGATTGTAGAAGGAGTAGCCTCGTG
7822	Cyan7822_3458	phage shock protein A (PspA) family protein	Cyan7822_3459	thioredoxin domain-containing protein	TAGTCAGCAATCAATAACAATCAGTGATACAGTTATCAGTGAACCAGAAACAGTTATCAGCGATAATGATTTACTGATTGGTAACGGGTGCAAGCAAGAGTGCCCTAAAAAACTGA**TAACTGA**CGACTGATTG---AAGGAGTAACCCCGTG
7424	PCC7424_2553	periplasmic solute binding protein	PCC7424_2554	ribosomal protein S4	>TAATGGACAATGGATAATTGATAATTGATAATGTAATCAAGACTTTAGCCATAAGAAACTTCAATTATTAATTAATATTGAAATAAATAAACAATAGAATAAATAAGTAAGTATCAATTATTCATTATCCATTCTCCATTATCCATTATCAATTATTT-ACAGTTATCAGCCCACCTAACGCA**TAACTGATAACTGATAACTGA**TTTA<
7822	Cyan7822_4235	cyclic pyranopterin monophosphate synthase subunit MoaA (EC 4.1.99.18)	Cyan7822_4234	SSU ribosomal protein S4P	>TGAAATTATTGATCTGATTGACTTAAACGGTTCAAAATCTCTAAAGTAATAGTTTAATTTGGCCGATAGTTTCTCTATATTTTTAATTTGAAAAAAAGCA--ACAGTTATCAGC-ACGAAGAACCA**TAACTGATAACTGATAACTGA**ATTA<
7424	PCC7424_4731	FeS assembly protein SufD	PCC7424_4732	cysteine desulfurase, SufS subfamily	TGAAAAATAGA**TAACTGATAACTGATAACTGATAACTGA**TGACTAA**TAACTGA**TAACTGTTAACTGTCAACTGATAACTAACAAATG
7822	Cyan7822_3141	Iron-regulated ABC transporter permease protein SufD	Cyan7822_3142	cysteine desulfurase, SufS subfamily	TAAAAATAGTCATTAGTCCTTAGTTTTTAGTAAATAATAAACTTTACTCAATCAACAAGTATTA**TAACTGA**CATAAAACTATTGACTAATGACCAATGACTAATAACCACTGACTCATGACGAAGAACAATG

Supplemental Table 5. TAACTGA repeats in *Flexibacter litoralis* DSM 6794 and *Paludibacter propionicigenes* WB4. Repeats are boldface (black for forward orientation, red for reverse), contiguous partial repeats blue. putative start and stop codons underlined.

**Upstream ORF**		**Downstream ORF**		**Sequence**
***Flexibacter litoralis* DSM 6794**				
Fleli_1962	hypothetical protein	Fleli_1963	dihydrodipicolinate reductase	TAAGAACGAGAGAATAGGGAATGCCAAAATTATTATACATTCAAAGTTATGCTGTAATTAGTTATTGAAATTAACTGGTTACTGA**TAACTGATAACTGATAACTGA**AATG
Fleli_2890	triosephosphate isomerase	Fleli_2891	Predicted helicase	TAAAATACATAT~310nt~TAAAATAATATTCACTCATTTACTGATTACTGGTAATTGA**TAACTGATAACTGA**ACATG
Fleli_0221	lipoate-protein ligase B	Fleli_0220	glutamine amidotransferase of anthranilate or aminodeoxychorismate synthase	TAGAATTACGA~78nt~AAGTTTTGAACTGA**TAACTGATAACTGATAACTGATAACTGATAACTGATAACTGATAACTGATAACTGATAACTGA**AAAATG
Fleli_0856	uracil-DNA glycosylase	Fleli_0855	hypothetical protein	TAACTTTGCG~54nt~TTTCATAA**TAACTGATAACTGA**TTTATG
Fleli_1748	hypothetical protein	Fleli_1749	asparagine synthase, glutamine-hydrolyzing	TAAACTAATTGA~132nt~TAACTAATTACTGGTAACTGCTCACTAA**TAACTGATAACTGA**AATTATG
Fleli_3312	anthranilate phosphoribosyltransferase	Fleli_3313	indole-3-glycerol phosphate synthase	TAAATTACGAA~70nt~TTTGATAACTGTTTACTGA**TAACTGATAACTGA**AAAAATG
Fleli_3902	hypothetical protein	Fleli_3901	ubiquitin-protein ligase	TAAGATAAAAGACA~135nt~TAGAATTTGACTAATTACTGTA**TAACTGATAACTGATAACTGA**AAAAATG
Fleli_2086	lycopene cyclase protein	Fleli_2085	DNA replication and recombinational repair protein RecR	(<2086)CATTGAATAATTTATGTAAAAA~158nt~AAATAATCATTTATGACAGATAACTACTTACTAA**TAACTGATAACTGA**TTAAAATG (2085>)
Fleli_3467	3-hydroxyacyl-CoA dehydrogenase	Fleli_3466	acetyl-CoA acetyltransferase	TAATAAATTCGAT~127nt~AAAACTGTTATCTATTTGGTTT**TAACTGA**TTGCTGG**TAACTGATAACTGA**AAAAATATG
Fleli_0439	acetyl-CoA carboxylase, biotin carboxyl carrier protein	Fleli_0440	acetyl-CoA carboxylase, biotin carboxylase subunit	TAATTTTAAATGATGA~161nt~TAGTATTGAATTAACTGGTAACTGGTTACTGA**TAACTGATAACTGA**TTAACGTTTG
Fleli_4030	hypothetical protein	Fleli_4029	1-acyl-*sn*-glycerol-3-phosphate acyltransferase	TAACAATCAACGTTA~90nt~TTAGATTTTATTAATTACAGTATTGATTACTATTCACTAG**TAACTGATAACTGA**TAACTGTTTACTGATAAAATAG
Fleli_3202	ribosomal protein L23	Fleli_3203	ribosomal protein L2	TAAAACTTTATTCTCTAAG~226nt~TTTTATTTACTGG**TAACTGA**TTACTGGTAATTGCTAACTGAATTGATAACTAATTACTGA**TAACTGATAACTGA**AGTTATTGTGCTGCAAAGCCTTTTTCGTCTAATCAAAATCAACATG
Fleli_3883	RNase HII	Fleli_3884	hypothetical protein (fragmented)	TAA**TCAGTTATCAGTTATCAGTTATCAGTTATCAGTTATCAGTTATCAGTTATCAGTTA**GGAAAAATTAAATTTCTAATATATTGTTTAAATAGTTTTTTTTTAAATAATTCATAATTATAAAAATG
Fleli_0210	near C terminus of PurC (SAICAR synthase) gene			ATG~928nt~TCTTTGAA**TCAGTTATCAGTTA**TCAAGAA**TCAGTTA**CCAGTTAA
***Paludibacter propionicigenes* WB4**				
Palpr_0520	pyruvate flavodoxin / ferredoxin oxidoreductase domain protein	Palpr_0519	thiamine pyrophosphate tpp-binding domain-containing protein	TAATAATGGCTATACAATTATCCGCTGAATGTTGTAGTTATTGCAACAT**TAACTGA**TCATTGA**TAACTGATAACTG**AAATATTATTATG
Palpr_0786	transcriptional regulator, Crp/Fnr	Palpr_0787	TonB-dependent receptor family	TGAACAATATCTCCTCTTCAAATTTTTTTT~295nt~CATATAGACTGA**TAACTGATAACTGATAACTGATAACTGA**AATCGGGAAGG~149nt~ATCGTCAAATAGTTACAACTGTG

Supplemental Table 6. TAACTGA repeats in *Elizabethkingia anophelis* NUHP1. All repeats are intergenic. Start and stop codons of flanking ORFs are underlined, and TAACTGA sequences are in boldface. The second two sequences are 98% identical; differences are highlighted in blue italics. The putative downstream proteins encoded by BD94_0852 and BD94_3031 are 96% identical over their first 966 aa, then diverging in the carboxyterminal ~220 amino acids. Those encoded by the upstream BD94_0853 and BD94_3030 are less similar, but share 51% identity over ~250 aa at the C-terminus of BD94_3030 (the shorter of the two).

Upstream ORF	Upstream description	Downstream ORF	Downstream description	Intergenic sequence
BD94_0853	hypothetical protein (1354 aa)	BD94_0852	RHS repeat-associated core domain (1190 aa)	TAAGTACGAAGTCGGAAGTTAGAATTACGAGGTACAAAATATTGAAATGAAGAGAGAAAGAAGCAGG***T***TGGTGCTTTCATCACCTGCCCTCTCTTTAATTAGAAGTTAGAAAC***T***AGAGGTTAGATTTTAGAAGCTAGCCCATATAGCCTCATACCTGAACCTTTGGTTGTTCTTATTACTAACCTTTC***A***AAAAAACACAAAAAAGCTAAAACTTTTTTTTAATACGTCAAGTTTTGACGCCTTCAGCAATGATCTTTGCTTCAAGATAAGAAGTTAGAGGTTATCGGAATAGGTTGA(TGGTTGAx3)**(*TAACTGA x4*)**TAACCGA(CAACTGAx3)**(TAACTGAx2)**TAACCGA(TAACTGGx2)**TAACTGA**TAACCACCAAACCATATAACTTACAACCTGTCAACCTTACAACTAAATCGCTGAAAAACAAAAACACAAAAAATCGCAAAAAGTAATTATTAATCTTTAAAAACCAAATCGTTATG
BD94_3030	hypothetical protein (803 aa)	BD94_3031	RHS repeat-associated core domain (1181 aa)	TAAGTACGAAGTCGGAAGTTAGAATTACGAGGTACAAAATATTGAAATGAAGAGAGAAAAAAGCAGG***C***TGGTGCTTTCATCACCTGCCCTCTCTTTAATTAGAAGTTAGAAAC***C***AGAGGTTAGATTTTAGAAGCTAGCCCATATAGCCTCATACCTGAACCTTTGGTTGTTCTTATTACTAACCTTTC_AAAAAACACAAAAAAGCTAAAACTTTTTTTTAATACGTCAAGTTTTGACGCCTTCAGCAATGATCTTTGCTTCAAGATAAGAAGTTAGAGGTTATCGGAATAGGTTGA(TGGTTGAx4)**(*TAACTGA x2*)**TAACCGA(CAACTGAx3)**(TAACTGAx2)**TAACCGA(TAACTGGx2)**TAACTGA**TAACCACCAAACCATATAACTTACAACCTGTCAACCTTACAACTAAATCGCTGAAAAACAAAAACACAAAAAATCGCAAAAAGTAATTATTAATCTTTAAAAACCAAATCGTTATG
BD94_0857	patatin family protein	BD94_0856	hypothetical protein	TAATGGTGAATAGTTGTTGGTTGTTGGTTGCCAGAATTGAAATCTTCCCATTTATTATCCCTTAAGAACACAGAAAAGCAAAAAAATTGAAAATAATTTTTAATGCGTCAAGTTCTGTCGCCACGGGTCAGGATCTTTGTACTGTTGATGGTTGACAGCTGACAACTGA**TAACTGATAACTGA**TAACCAACAACTCACTCCCCTTCTCATTTAAAACCTTACGCCTATGAACATCACGAAATTGCCGTAACAGGCAATTACAAAAATCAATTCATTACTAACCTTAAAATAAAATATTATG

Supplemental Table 7. XisH and XisI annotations and BOGUAY_0693 orthologs in species with TAACTGA repeats. Updated from (MacGregor et al., 2013).

	XisH annotations	XisI annotations	BOGUAY_0693 orthologs
***Beggiatoaceae* (GAMMAPROTEOBACTERIA)**			
Cand. "Thiomargarita nelsonii"	5	7	11
Orange Guaymas "Maribeggiatoa"	11	12	29
Thioploca ingrica	0	0	5
Beggiatoa alba	0	1	1
***Chromatiaceae* (GAMMAPROTEOBACTERIA)**			
Thiocystis violascens DSM 198	0	0	0
**CYANOBACTERIA**			
Chroococcidiopsis thermalis PCC 7203	2	5	0
Microcystis aeruginosa NIES-843	6	8	0
Calothrix PCC 7507	17	21	11
"Nostoc (Anabaena) azollae" 0708	1	2	1
Stanieria cyanosphaera PCC 7437	0	0	0
Calothrix PCC 6303	10	9	2
Nodularia spumigena CCY9414	3	7	1
Rivularia sp. PCC 7116	2	4	5
Pleurocapsa sp. PCC 7327	0	1	0
Cyanothece PCC 8801	2	4	2
Halothece sp. PCC 7418	3	3	1
Cyanothece PCC 8802	3	4	2
Nostoc sp. PCC 7107	3	8	3
Crinalium epipsammum PCC 9333	6	7	2
Cyanothece PCC 7424	2	4	1
Cyanothece PCC 7822	4	5	2
**BACTEROIDETES**			
Flexibacter litoralis DSM 6794	0	0	1
Elizabethkingia anophelis NUHP1	0	0	0
Paludibacter propionicigenes WB4	0	0	0
Gramella forsetii KT0803	0	0	0
Aequorivita sublithincola DSM 14238	0	0	0

Supplemental Table 8. Partial census of predicted Shine-Dalgarno sequences in the BOGUAY genome. Sequences shown were counted if their 3′-most unambiguous base occurred between 4 and 13 nt upstream of a predicted start codon. If two possible SD sequences were present, the one closer to the start codon was counted. Where two of different length overlapped, the longer one was counted. Where only part of the genome was surveyed, it was the same part in all cases (IMG Gene IDs 2502843947-2502842473). The total genome contains 5272 predicted protein-coding genes; the possible SD sequences here account for an estimated 1346 of these. Ambiguity codes are B: not A, V: not U, H: not G, and N: any.

Sequence	Occurrences counted	Proportion of genome surveyed	Total or estimated total occurrences as possible SD
**AGGAGGU**	19	1	19
B**GGAGGU**	18	1	18
**AGGAGG**V	44	1	44
**AGGAG**HN	98	0.28	350
B**GGAGG**V	15	0.28	54
NH**GAGGU**	33	0.28	118
**AGGA**HNN[1]	146	0.28	522
B**GGAG**HN	35	0.28	125
NH**GAGG**V[1]	23	0.28	100
NNH**AGGU**	69	0.28	246

[1]33 upstream regions contained the sequence "GAGGA", which was arbitrarily counted as "AGGA" but could also be "GAGG".

Supplemental Table 9. First 100 blastp results for the sequence SVISYQLSV. Repeat units and partial repeats are separated by spaces and highlighted in **bold.**

Accession number	Strain	Description	Most consecutive repeats	Total complete repeats	Sequence
EDN72534.1	*Beggiatoa* sp. SS	hypothetical protein	2	2	MAFYHQLST **VISYQLS VISYQLS V** NR **YQLS** IISRSKRRLKKGYQ
EDN72750.1	*Beggiatoa* sp. SS	hypothetical protein	2	2	MFFIVYSII **SVISYQL SVISYQL SVI** NEPLSKYLNLSKALSKLHTLLKKAS
EDN72971.1	*Beggiatoa* sp. SS	hypothetical protein	2	2	MLHSF **QLSVISY QLSVISY QLSV** LFHPGFDFHFSLYNNEC
EDN72777.1	*Beggiatoa* sp. SS	hypothetical protein	2	2	MNERHV **SYQLSVI SYQLSVI SYQL** LFNIATKCASGRSVLKLKKN
EDN72295.1	*Beggiatoa* sp. SS	hypothetical protein	4	4	MFNSKRRRQVERNKTPHNSKKSPKAVKAKGLFFF **SYQLSVI SYQLSVI SYQLSVI SYQLSVI SYQLS** LH
AFY30559.1	*Calothrix* sp. PCC 7507	hypothetical protein	2	2	MVLIVKSSLVKT **VISYQLS VISYQLS V** TGTENLDLARWNQSRSIGYQVNC
WP_042341192.1	*Calothrix* sp. PCC 7507	hypothetical protein	4	4	MFSLLVNFTGLNQLAHLASGIRVN **SYQLSVI SYQLSVI SYQLSVI SYQLSVI SYQLSV** ERMY
CCQ52685.1	*Crocosphaera watsonii* WH 8502	hypothetical protein	2	2	MLSGFSGLLQLGLRQYSIFF **SYQLSVI SYQLSVI S** DH
ELS44702.1	*Microcystis aeruginosa* DIANCHI905	hypothetical protein	1	1	MIYRKNCFLSRFLAFVFSSAKPTDYKLSFPVIRSLVKFSRTPS **SVISYQL SV** SRFEFSVA
ELS50041.1	*Microcystis aeruginosa* DIANCHI905	hypothetical protein	1	1	MQTF **SVISYQL SVIS** CQLSVV **SYQL SVI** RCEFLVYCLLFT
ELS50052.1	*Microcystis aeruginosa* DIANCHI905	hypothetical protein	1	1	MSCGDSALAFASHFD **SVISYQL SVIS** GEMGKWGDGEVGKWGSGEKGE
ELS45238.1	*Microcystis aeruginosa* DIANCHI905	hypothetical protein	1	1	MSLHLAPHRGLPSQYLSILLVRSYRTFAPLPP **ISYQ** V **SVISYQL SV** ESYQWKVISYQ
ELS47528.1	*Microcystis aeruginosa* DIANCHI905	hypothetical protein	1	1	MSPGKTAKIANQYGQIVDNALI **QLSVISY QLSV** T **SYQLSV** T **SYQLSV** T **SYQL** PV **ISYQLS** R
ELS45220.1	*Microcystis aeruginosa* DIANCHI905	hypothetical protein	1	1	MSSTVK **QLSVISY QLSV** RTCWGFGVLVEISPSPYHLTSVL
ELS45851.1	*Microcystis aeruginosa* DIANCHI905	hypothetical protein	1	1	MTSLV **QLSVISY QLSV** FFSFQRLAFGVRLFEQMVFDDPILLLMRSKLTAAGLKIPVT
ELS44871.1	*Microcystis aeruginosa* DIANCHI905	hypothetical protein	2	2	MDVLAK **SVISYQL SVISYQL** FSSLFPSPFSLFPNTYSFKDYV
ELS48290.1	*Microcystis aeruginosa* DIANCHI905	hypothetical protein	2	2	MTTAAPNPFKIFPPF **QLSVISY QLSVISY Q** WEVGSGEVGRINKNSLPSPVSESPDRFKMADSLIFTVHRQLF
ELS45017.1	*Microcystis aeruginosa* DIANCHI905	hypothetical protein	4	4	MVAWLWLN **SVISYQL SVISYQL SVISYQL SVISYQL SVISYQ** IKVISFRF
GAL91692.1	*Microcystis aeruginosa* NIES-44	hypothetical protein	1	1	MFSSRVIQRTFWTVARLSFKTATG **LSVISYQ LSVIS** G
GAL91279.1	*Microcystis aeruginosa* NIES-44	hypothetical protein	1	1	MLIFVQHWVRSQE **SVISYQL SVIS** SLRKLPTPYTPHPTPYTLHPTPYTPLYFNLTAIIAKL
GAL94955.1	*Microcystis aeruginosa* NIES-44	hypothetical protein	1	1	MLLKKHGRGKFDPS **VISYQLS VISYQL** PVFSFQLP **VISYQ** M
GAL92691.1	*Microcystis aeruginosa* NIES-44	hypothetical protein	1	1	MTSLVQF **SVISYQL SV** FFGFQRLAFGVQFFEQMVFDDPILLLMRSKLTAASLKIPVP
GAL95234.1	*Microcystis aeruginosa* NIES-44	hypothetical protein	1	1	MVI **SVISYQL SVIS** D **YQ** QSLVAFNFHSLARGDLLPLVFGHI
GAL95746.1	*Microcystis aeruginosa* NIES-44	hypothetical protein	1	1	MSCGDSALGFASHFD **SVISYQL SVISYQ** WGDGGVGCGEVGKWGSGEKGE
GAL94774.1	*Microcystis aeruginosa* NIES-44	hypothetical protein	1	2	MEHG **QLSVISY QLSV** GKLT **VISYQLS V** GKLTVIRFEF
GAL95315.1	*Microcystis aeruginosa* NIES-44	hypothetical protein	1	2	MPSSHN **QLSVISY QLSV** GKLSVGK **LSVISYQ LSV** GKLSVGKLSDLSFK

GAL91354.1	*Microcystis aeruginosa* NIES-44	hypothetical protein	2	2	ME **ISYQLSV ISYQLSV** GCGVWGVGCGVWGEQSCLLHECLLHECLLHECLLPPVSCLD
GAL95119.1	*Microcystis aeruginosa* NIES-44	hypothetical protein	2	2	MIHESTLPVGGVARPLR **SVISYQL SVISYQL SVISY** H
GAL94023.1	*Microcystis aeruginosa* NIES-44	hypothetical protein	2	2	MIPPNLHPIRLVAGKF **SYQLSVI SYQLSVI S** FFLPSP
GAL94328.1	*Microcystis aeruginosa* NIES-44	hypothetical protein	2	2	MKSVAF **SVISYQL SVISYQL** LGLSFKLLGLSFKLLGLSY
GAL91906.1	*Microcystis aeruginosa* NIES-44	hypothetical protein	2	2	MLIFFKKYNSCKNPTSTIGLEV **SYQLSVI SYQLSVI SYQL** SVH
GAL94407.1	*Microcystis aeruginosa* NIES-44	hypothetical protein	2	2	MNGDCK **SVISYQL SVISYQL** EKARVFINLSSHIFHSLILSPVS
GAL93711.1	*Microcystis aeruginosa* NIES-44	hypothetical protein	2	2	MSSRNCIKNRPSCSKIAKATNI **ISYQLSV ISYQLSV** FGIFLAGF
GAL95073.1	*Microcystis aeruginosa* NIES-44	hypothetical protein	2	2	MTANISGRSIHLSFIIYHL **SVISYQ** S **SVISYQL SVISYQL SVIS** KVMGGLGTGVWSSFLSPYPLLFPNPYS
GAL94647.1	*Microcystis aeruginosa* NIES-44	hypothetical protein	2	2	MTLTVRR **QLSVISY QLSVISY Q** FTVYCLLITEKLPTSPLPNL
GAL94617.1	*Microcystis aeruginosa* NIES-44	hypothetical protein	2	3	MVANRFEIRHN **SVISYQL SVIS** H **QLSVISY QLSVISY Q** IGVLSEQYVLSEQY
GAL92340.1	*Microcystis aeruginosa* NIES-44	hypothetical protein	3	3	MAIEHG **YQLSVIS YQLSVIS YQLSVIS YQ** WEVGSGKWEVGSGKWEVGSGKWGDHCVCVAGGEWGI
GAL95642.1	*Microcystis aeruginosa* NIES-44	hypothetical protein	3	3	MALVGECLSLVAGVRGFF **SYQLSVI SYQLSVI SYQLSVI SYQL** L **VIS**
GAL91365.1	*Microcystis aeruginosa* NIES-44	hypothetical protein	3	3	MARLN **QLSVISY QLSVISY QLSVISY QLS** DYSFKWTVLGENFANFFSLITVH
GAL91727.1	*Microcystis aeruginosa* NIES-44	hypothetical protein	3	3	MDSRLIIF **SYQLSVI SYQLSVI SYQLSVI SYQLSV** LSGNCGAFFFLFSDYCLLITDY
GAL95729.1	*Microcystis aeruginosa* NIES-44	hypothetical protein	3	3	MVVLVSISGSGNDSN **QLSVISY QLSVISY QLSVISY QLS** INLVLFAVH
GAL91403.1	*Microcystis aeruginosa* NIES-44	hypothetical protein	4	4	MRGGWGIWHF **QLSVISY QLSVISY QLSVISY QLSVISY QL** PVTSYQI
BAG05892.1	*Microcystis aeruginosa* NIES-843	unknown protein	1	1	MAKTNKRVTKTRVWGASLCQKPKFWHSFSQEKVAICSIY **SVISYQL SV** RKGI
BAG00781.1	*Microcystis aeruginosa* NIES-843	unknown protein	1	1	MF **SVISYQL SVISY** SRRQESGESVPMKKFSPPQRKEVSFPTPHTPHPTPPFKSGVES
BAG02424.1	*Microcystis aeruginosa* NIES-843	unknown protein	2	2	M **ISYQLSV ISYQLSV ISYQ** GNKGTRAIGKSITNFPPVSCLLSPVSYLLTI
BAG05579.1	*Microcystis aeruginosa* NIES-843	unknown protein	2	2	MFSSRVIQRTFWTVARLSFKTATG **LSVISYQ LSVISYQ** WVSYQLSGKFQLKPQNPKNPTT
BAG05227.1	*Microcystis aeruginosa* NIES-843	unknown protein	2	2	MRNVNLNILKFLESINNFCLSLSNMGLEV **SYQLSVI SYQLSVI SYQLSV** H
BAG01649.1	*Microcystis aeruginosa* NIES-843	unknown protein	3	3	MSGDRR **QLSVISY QLSVISY QLSVISY QL** WFFGLCYAMSFSIRVSSLIEKDIDAIFVNCFRSRTN
CAO89605.1	*Microcystis aeruginosa* PCC 7806	unnamed protein product	1	1	MSLHLAPHRGLPSQYLSILLVRSYRTFAPLPP **ISYQ** V **SVISYQL SV** ESYQWKVISYQ
CAO89377.1	*Microcystis aeruginosa* PCC 7806	unnamed protein product	2	3	MMFYKECRPTKLKKRPNYALSILGFRQF **QLSVISY QLSVISY QLSVIS** SLFTVYCLLTTEKTTHSPTPWLF
CAO89567.1	*Microcystis aeruginosa* PCC 7806	unnamed protein product	3	3	MFLLGNWVGSF **QLSVISY QLSVISY QLSVISY QLSVIS** DYHWSSVA
CAO87942.1	*Microcystis aeruginosa* PCC 7806	unnamed protein product	4	4	MRKLLSLLFSPKKPKVNPLN **QLSVISY QLSVISY QLSVISY QLPVISY QLSV** T **SYQLSV** R
CCI09800.1	*Microcystis aeruginosa* PCC 7941		2	2	MNPATANYDEPWKEALSEYFEAFLYFFFPEVHQLISN **QLSVISY QLSVISY Q**

CCI05885.1	*Microcystis aeruginosa* PCC 7941	conserved hypothetical protein	3	3	MFW **SVISYQL SVISYQL SVISYQL SVI** RCEFLVYCLLFTGYWLLFTGYWLLFNWLLFTVYCLLFTGYCLTV
CCI08979.1	*Microcystis aeruginosa* PCC 7941	conserved hypothetical protein	4	4	MRNVNLNILKFLESMNNFCLSLSNIELGDRS **QLSVISY QLSVISY QLSVISY QLSVISY QLSV** N
WP_043998322.1	*Microcystis aeruginosa* PCC 9432	hypothetical protein	2	2	MCFG **QLSVISY QLSVISY QLSVI** RCEFLVYCLLFTVYCLLFTGYWLLFTGYWLLVTELISPTEDNPTPS
CCH92894.1	*Microcystis aeruginosa* PCC 9432	conserved hypothetical protein	2	2	MSVH **LSVISYQ LSVISYQ LS** DLSFKFTDLPAPHTPHPAPHFPN
CCI01690.1	*Microcystis aeruginosa* PCC 9443	hypothetical protein	1	1	MAGISDQLSVSSD **QLSVISY QLSVIS** DQGVGEWGNSTNTLKPQHPNPQHDC
CCI02938.1	*Microcystis aeruginosa* PCC 9443	conserved hypothetical protein	1	3	MRNVNLNILKFLESVDNFCLSLSNIGLEV **SYQLSVI S** E **QLSVISY QLSVIS** E **QLSVISY Q**
WP_043997014.1	*Microcystis aeruginosa* PCC 9443	hypothetical protein	2	2	M **SVISYQL SVISYQL SV** T **SYQLS** REAQLFVGWVSGSVTHAGVGFHASTQPTFILYLISPTH **LSVIS** DSVTSYQM
CCI05012.1	*Microcystis aeruginosa* PCC 9443	hypothetical protein	4	4	MPPLRERLLILISLWALVKLILAVTAVKLSSK **QLSVISY QLSVISY QLSVISY QLSVISY Q** WKA
WP_043996333.1	*Microcystis aeruginosa* PCC 9443	hypothetical protein	4	8	M **SYQLSVI SYQLSVI SYQLSVI SYQLSVI SYQ** W **SVISYQL SV** V **SYQLSVI SYQLSVI SYQLSVI S** GQ
CCI37464.1	*Microcystis aeruginosa* PCC 9701	GP63-like protein (fragment)	1	1	MLATETRVQVSILGYLFSDLKEAVGGRQD **SVISYQL SV** H
CCH99635.1	*Microcystis aeruginosa* PCC 9717	hypothetical protein	3	3	MMFYKECRATKLKKRPNYAPSILGFRQFQLL **VISYQL** L **VISYQLS VISYQLS VISYQLS** GDYFYLFSPVHNS
CCI14658.1	*Microcystis aeruginosa* PCC 9806	conserved hypothetical protein	1	1	MIDWTKIPESLEKELKRITASAKTKKRFADKLY **SVISYQL SVIS** D
CCI12155.1	*Microcystis aeruginosa* PCC 9806	conserved hypothetical protein	1	2	MAGISH **QLSVISY QLSVIS** D **QLSVISY Q** PSVNRDIGRKKLMADG
CCI13699.1	*Microcystis aeruginosa* PCC 9806	hypothetical protein	2	2	MLIFAQHWVRS **QLSVISY QLSVISY QL** TEKTPHTPHPTPHTLHPTPYTPHPTPHTLHPTPYTPHPTPHTLHPVP
CCI14987.1	*Microcystis aeruginosa* PCC 9806	conserved hypothetical protein	2	2	MQKRESNPNILRSL **ISYQLSV ISYQLSV** TSYQLSGLSFKLSS
CCI14290.1	*Microcystis aeruginosa* PCC 9806	hypothetical protein	2	2	MSVFLQMWDTPDFDCMKLRYS **SYQLSVI SYQLSVI SYQLSV** LNSSFPLSLH
CCI13897.1	*Microcystis aeruginosa* PCC 9806	hypothetical protein	3	3	MGLGSF **SYQLSVI SYQLSVI SYQLSVI SYQL** LVFRFELLVHCLLTTEKTPTPPHL
WP_044000143.1	*Microcystis aeruginosa* PCC 9806	hypothetical protein	5	5	MSIIAKLATIIN **SYQLSVI SYQLSVI SYQLSVI SYQLSVI SYQLSVI SYQL** EGDRKMGE
WP_044000126.1	*Microcystis aeruginosa* PCC 9806	hypothetical protein	6	6	M **SYQLSVI SYQLSVI SYQLSVI SYQLSVI SYQLSVI SYQLSVI SYQLS** GLIFKLLGLSY
CCI17482.1	*Microcystis aeruginosa* PCC 9807	hypothetical protein	2	2	MAFSSPLAISLLPVHRPDFFFLNPF **ISYQLSV ISYQLSV ISYQL** LVLSFKFSDHCLLITENTSSFIIHNS
CCI17311.1	*Microcystis aeruginosa* PCC 9807	hypothetical protein	2	2	MALI **QLSVISY QLSVISY QLS** DVSFQFNDYCLLIKVSSKVSP
CCI20175.1	*Microcystis aeruginosa* PCC 9807	modular protein	2	2	MNQTTANYDEPWKEALTEYFESFLYFFFPEAHQL **ISYQLSV ISYQLSV** TNWKQVSG
CCI19235.1	*Microcystis aeruginosa* PCC 9807	conserved hypothetical protein	2	3	M **ISYQLSV IS** D **QLSVISY QLSVISY QLSVI** TSLRKLPTPHTPHPTPHPLFDF
CCI17323.1	*Microcystis aeruginosa* PCC 9807	hypothetical protein	3	3	MLIVYRWWGKN **VISYQLS VISYQLS VISYQLS VIS** LLTTEINSAYFLGLIFWGFI
CCI14850.1	*Microcystis aeruginosa* PCC 9807	conserved hypothetical protein	4	4	M **ISYQLSV ISYQLSV ISYQLSV ISYQLSV ISYQLS** DLSFQFTVYCLRKNPRKL

CCI17213.1	*Microcystis aeruginosa* PCC 9807	conserved hypothetical protein	4	4	MRNVNLNILKFLYSINKFYLSFTNIGLEV **SYQLSVI SYQLSVI SYQLSVI SYQLSVI SYQL** TEKTPHTPHPTPH
CCI19002.1	*Microcystis aeruginosa* PCC 9807	conserved hypothetical protein	5	5	MPVYSPVVNL **SYQLSVI SYQLSVI SYQLSVI SYQLSVI SYQLSVI SYQ** M
CCI24337.1	*Microcystis aeruginosa* PCC 9808	hypothetical protein	2	2	MLLKKYGRGKFDP **SVISYQL SVISYQL** PVTSFQFSVT **SYQLS** DVSFQFTDYCLLFTGRLRRAFGNTEKN
CCI26609.1	*Microcystis aeruginosa* PCC 9808	hypothetical protein	2	3	M **SVISYQL SVISYQL** L **VISYQLS** DVSCQLLASCMSACPYK
CCI26719.1	*Microcystis aeruginosa* PCC 9809	hypothetical protein	1	1	MISD **QLSVISY QLSV** HFLLFTDITFWAHAMRLYHIKTD
CCI22702.1	*Microcystis aeruginosa* PCC 9809	hypothetical protein	2	2	MIKTTRKIA **SVISYQL SVISYQL** TVYSKKLPTSQLIIHNL
CCI25786.1	*Microcystis aeruginosa* PCC 9809	hypothetical protein	2	2	MPPLRDRLLILISLRALVKPILAVTAVKLISN **QLSVISY QLSVISY QLSV** ESSWLKAIIQESTAYQ
CCI26345.1	*Microcystis aeruginosa* PCC 9809	conserved hypothetical protein	3	3	MSCQEIGD **SYQLSVI SYQLSVI SYQLSVI SYQL** WFFGLCYAMSFSIRDETLIEKDIDAIFVNCFRSRTN
WP_036397446.1	*Microcystis aeruginosa* SPC 777	hypothetical protein	7	7	MGFN **QLSVISY QLSVISY QLSVISY QLSVISY QLSVISY QLSVISY QLSVISY QLSV** ELL
ELP53317.1	*Microcystis aeruginosa* TAIHU98	hypothetical protein	1	1	M **SVISYQL SVIS** FESERGTIPLNALSTKVLKCQPLRGSGGKFLDFFPEN
ELP54722.1	*Microcystis aeruginosa* TAIHU98	hypothetical protein	1	1	MSIAEKTDRSH **YQLSVIS YQLSV** HPLLFTEKRLPTSPPPHFPTSPIHNS
ELP53993.1	*Microcystis aeruginosa* TAIHU98	hypothetical protein	2	2	MAFSSPLAISLLPVDQPDFFLKPLY **QLSVISY QLSVISY**
ELP54385.1	*Microcystis aeruginosa* TAIHU98	hypothetical protein	2	3	MKQTTANYDEPWKEALTEYFEAFLHFFFPEVHQL **ISYQLSV ISYQLSV** T **SYQLSVI S** D
ELP53443.1	*Microcystis aeruginosa* TAIHU98	hypothetical protein	3	3	MWKSAKT **VISYQLS VISYQLS VISYQLS V** PRKRQFST
CCI31614.1	*Microcystis* sp. T1-4	conserved hypothetical protein	1	2	MPTY **LSVISYQ LSVIS** HQFTDYCLLFTVHCSLKIPPT **ISYQLSV ISYQ** M
CCI31613.1	*Microcystis* sp. T1-4	hypothetical protein	2	2	MSCQFTDYCLLFTVHCSLKIPPT **ISYQLSV ISYQLSV I** RCELSVH
CCI32135.1	*Microcystis* sp. T1-4	hypothetical protein	2	3	MLVGFFIAV **SYQLSVI SYQLSVI S** N **QLSVISY** GSSVCPMQWRGL
WP_039900645.1	*Microcystis* sp. T1-4	hypothetical protein, partial	3	6	M **SYQLS** GV **SYQLSVI SYQLSVI SYQLSVI SYQLS** GV **SYQLS** GV **SYQLSVI SYQLSVI SYQ** E **SVISYQL S** GVS
CCI31776.1	*Microcystis* sp. T1-4	conserved hypothetical protein	4	4	MF **SVISYQL SVISYQL SVISYQL SVISYQL SV** TLGVRSQEAGVESWGFRVLGF
CCI30340.1	*Microcystis* sp. T1-4	hypothetical protein	5	5	MIIRTKSLVILKKKQLKIMLVDVLAK **SVISYQL SVISYQL SVISYQL SVISYQL SVISYQL SV**
WP_044494194.1	*Moorea producens*	hypothetical protein	2	2	MSLLRKLSAISYQLSAISYYLSVISYQLSVII **YQLS** A **ISY** Y **LSVISYQ LSVISYQ LS** GFSFQLMG
WP_044493786.1	*Moorea producens*	hypothetical protein	3	4	MEKSPNFSTTFSTNGLLAQLVF **SYQLS** A **ISYQLSV ISYQLSV ISYQLS** AISH **QLSVISY QLS** G **ISYQLS** AIS
WP_039714530.1	*Scytonema millei*	hypothetical protein, partial	1	1	MVAFTASLSRA **VISYQ** R **SVISYQL SV** NRQPSTANRQPSTANRQPSTANRQLSTVNRQPST

158

Supplemental Table 10. BOGUAY ORFs containing CSP-CDS domains. For a review of conserved sequences see (Horn et al., 2007). 1HZA_B is Chain B, *Bacillus caldolyticus* cold-shock protein, used by CDD to define the domain. Protein domains identified by CDD (Marchler-Bauer et al., 2011) and subcellular localization predicted by PSORTb 3.0 (Yu et al., 2010) are shown in parentheses after the protein descriptions. CSP_CDS stands for Cold-Shock Protein with an S1-like cold-shock domain (CSD); Excalibur domains are defined as extracellular calcium-binding domains similar to eukaryotic EF-hand domains (Rigden et al., 2003), "extracellular" because of the proteins they were originally identified in. Start codons, stop codons, and possible ribosome binding sites are underlined. No TAACTGA repeats were identified.

Contig_ORF	Putative function	Predicted domain structure	Predicted RNA-binding motif (RNP1-loop-RNP2)	Predicted cellular location	Upstream DNA sequence to next ORF
01308_0439	Major cold shock protein CspA	CSP_CDS	18 GYGFIER DGGNEPD VFVH 35	cytoplasmic, match with CSPB_GEOSE	<CATAGATTTCTAATTTTTGCTAAGTAAG TTATGCCAGCTTTATCAAAAGGCTGGGTT TTAATAAAAAAGCCAATCTAGGTTGGCAC ATTTAAGTGATGAGGTTTATATG>
00024_0708	Cold shock protein CspE	CSP_CDS	14 GFGFISP EDGSKD VFVH 30	cytoplasmic, match with DCSPD_ECOLI	TAA~640nt~TTATTTAAAGTCTTATTTC AGATTTTTTTTATTTAGTGATTTATCCAA CTTAAAAAGTTGGTTTTAGATTGACATAT TGACATAATGAGGTTAAATTGGTATG
01318_2110	Cold-shock DNA-binding domain protein	CSP_CDS – Excalibur	24 GFGFILP ENCGKN IFIH 40	unknown	(beginning of contig) TTAATATG>
00441_3761	Cold-shock DNA-binding domain protein	CSP_CDS – Excalibur	15 GFGFIRT KELPKD VFVH 31	unknown	<CATGGTATAGTTTTTCTATCTAATAGTT ATTATCTATGCTAATTGTATTGTATTGAA TTGTACAGATTTCAAGTAATCATTGCTTT ATATTCCGAAGCTTATGTAATATTTTTTA TACTATATTTCAATTGTTGAGGTGAAAAA AAACATG>
00322_3107	(none assigned)	CSP_CDS-DUF1294	15 GFGFINS KTGMPD VFIH 31	cytoplasmic membrane, because four internal helices found	TAA~334nt~AAATTCAAACAACAAAAAG CTTAACTTTGCGCTGAAGCCTTTGCTGTA AAGAAAGACCGCGTGGTTTAGCTTGATCG TTAGGTATTCATAAAATAAGCTCATG
1HZA_B			GYGFIEV EGGSD VFVH		

References

Horn, G., Hofweber, R., Kremer, W., and Kalbitzer, H.R. (2007). Structure and function of bacterial cold shock proteins. *Cellular and Molecular Life Sciences* 64**,** 1457-1470.

MacGregor, B.J., Biddle, J.F., and Teske, A. (2013). Mobile elements in a single-filament orange Guaymas Basin *Beggiatoa* (*Maribeggiatoa*) sp. draft genome: Evidence for genetic exchange with cyanobacteria. *Applied and Environmental Microbiology* 79**,** 3974-3985.

Marchler-Bauer, A., Lu, S.N., Anderson, J.B., Chitsaz, F., Derbyshire, M.K., Deweese-Scott, C., Fong, J.H., Geer, L.Y., Geer, R.C., Gonzales, N.R., Gwadz, M., Hurwitz, D.I., Jackson, J.D., Ke, Z.X., Lanczycki, C.J., Lu, F., Marchler, G.H., Mullokandov, M., Omelchenko, M.V., Robertson, C.L., Song, J.S., Thanki, N., Yamashita, R.A., Zhang, D.C., Zhang, N.G., Zheng, C.J., and Bryant, S.H. (2011). CDD: a Conserved Domain Database for the functional annotation of proteins. *Nucleic Acids Research* 39**,** D225-D229.

Rigden, D.J., Jedrzejas, M.J., and Galperin, M.Y. (2003). An extracellular calcium-binding domain in bacteria with a distant relationship to EF-hands. *FEMS Microbiol Lett* 221**,** 103-110.

Yu, N.Y., Wagner, J.R., Laird, M.R., Melli, G., Rey, S., Lo, R., Dao, P., Sahinalp, S.C., Ester, M., Foster, L.J., and Brinkman, F.S.L. (2010). PSORTb 3.0: improved protein subcellular localization prediction with refined localization subcategories and predictive capabilities for all prokaryotes. *Bioinformatics* 26**,** 1608-1615.
